# Integrating anoikis and ErbB signaling insights with machine learning and single-cell analysis for predicting prognosis and immune-targeted therapy outcomes in hepatocellular carcinoma

**DOI:** 10.3389/fimmu.2024.1446961

**Published:** 2024-10-11

**Authors:** Huipeng Fang, Xingte Chen, Yaqi Zhong, Shiji Wu, Qiao Ke, Qizhen Huang, Lei Wang, Kun Zhang

**Affiliations:** ^1^ Department of General Surgery, Affiliated Fuzhou First Hospital of Fujian Medical University, Fuzhou, China; ^2^ Department of Radiation Oncology, Clinical Oncology School of Fujian Medical University, Fujian Cancer Hospital, Fuzhou, China; ^3^ Department of Hepatopancreatobiliary Surgery, The Cancer Hospital of the University of Chinese Academy of Sciences (Zhejiang Cancer Hospital), Hangzhou, China; ^4^ Department of Radiation Oncology, Mengchao Hepatobiliary Hospital of Fujian Medical University, Fuzhou, China; ^5^ Department of Radiation Oncology, Jiangxi Clinical Research Center for Cancer, Jiangxi Cancer Hospital, The Second Affiliated Hospital of Nanchang Medical College, Nanchang, Jiangxi, China

**Keywords:** hepatocellular carcinoma, anoikis, ErbB signaling, prognostic model, single cell analysis, machine learning

## Abstract

**Background:**

Hepatocellular carcinoma (HCC) poses a significant global health challenge due to its poor prognosis and limited therapeutic modalities. Anoikis and ErbB signaling pathways are pivotal in cancer cell proliferation and metastasis, but their relevance in HCC remains insufficiently explored.

**Methods:**

This study evaluates the prognostic significance of anoikis and ErbB signaling pathways in HCC by utilizing data from The Cancer Genome Atlas (TCGA), the International Cancer Genome Consortium (ICGC), three additional independent validation cohorts, and an in-house cohort. Advanced bioinformatics analyses and 167 machine learning models based on leave-one-out cross-validation (LOOCV) were used to predict HCC prognosis and assess outcomes of immune-targeted therapies. Additionally, key biological processes of the anoikis and ErbB signaling pathways in HCC were further investigated.

**Results:**

The single sample Gene Set Enrichment Analysis revealed a strong correlation between upregulated ErbB signaling in high anoikis-expressing tumors and poor clinical outcomes. The development of the Anoikis-ErbB Related Signature (AERS) using the LASSO + RSF model demonstrated robust predictive capabilities, as validated across multiple patient cohorts, and proved effective in predicting responses to immune-targeted therapies. Further investigation highlighted activated NOTCH signaling pathways and decreased macrophage infiltration was associated with resistance to sorafenib and immune checkpoint inhibitors, as evidenced by bulk and single-cell RNA sequencing (scRNA-seq).

**Conclusion:**

AERS provides a novel tool for clinical prognosis and paves the way for immune-targeted therapeutic approaches, underscoring the potential of integrated molecular profiling in enhancing treatment strategies for HCC.

## Introduction

Hepatocellular carcinoma (HCC), the most prevalent form of primary liver cancer, accounts for 75%–90% of cases and ranks as the sixth most common malignancy globally (1). It also represents the third-leading cause of cancer-related mortality, with a bleak 5-year survival rate of less than 20% (2, 3). Although advancements in treatments such as chemoembolization, targeted therapies (e.g., sorafenib and lenvatinib), and immune checkpoint blockade (ICB) have extended survival in patients with advanced HCC, overall outcomes remain generally poor (4–7). There is an urgent need to enhance current treatments and explore new therapeutic options. The potential of circulating tumor cells and nucleic acids, along with microvascular invasion, shows promise as biomarkers for HCC management, yet their clinical utility remains constrained by current detection technologies (8, 9). Therefore, discovering new biomarkers and therapeutic targets is essential to improve patient outcomes in HCC.

Members of the ErbB tyrosine kinase family such as EGFR and HER2 are frequently altered in cancer, driving tumor growth and progression through aberrant signaling pathways (10). In HCC, overexpression or dysregulation of ErbB receptors is commonly observed, correlating with poor prognosis and aggressive tumor behavior (11). Activation of the EGFR pathway stimulates downstream signaling cascades, such as Ras/Raf/MAPK and PI3K/Akt/mTOR, promoting cell proliferation and survival (12). Additionally, amplification or mutation contributes to HCC progression via dysregulated signaling (13). To combat this, targeted therapies have been developed, including tyrosine kinase inhibitors (TKIs), monoclonal antibodies (mAbs), and antibody–drug conjugates (ADCs), which specifically inhibit ErbB signaling (14). These therapies have demonstrated clinical efficacy, improving survival rates in patients with cancers that exhibit these genetic alterations (15). However, achieving lasting clinical benefits remains challenging due to resistance mechanisms that diminish the efficacy of these targeted therapies (16). This highlights the ongoing need for more effective treatment strategies in HCC, focusing on overcoming resistance and enhancing the durability of therapeutic responses to ErbB-targeted interventions.

Anoikis, a form of apoptosis triggered by cell detachment from the extracellular matrix, is vital for maintaining tissue integrity and suppressing tumorigenesis (17). However, cancer cells can evade anoikis, facilitating proliferation and metastasis (18). In HCC, understanding anoikis resistance has become increasingly attractive. Studies have shown that modulation of the mTOR/S6K1 signaling axis and the EGFR pathway influences HCC metastasis by regulating anoikis (19, 20). Various targets have been identified to mitigate anoikis resistance and inhibit HCC progression (21). For example, histidine-rich calcium-binding protein (HRC) and autophagy pathways have been implicated in promoting anoikis resistance and metastasis in HCC (22, 23). Despite these advances, further research is needed to develop prognostic models based on anoikis-related genes (ARGs) for improved HCC prognosis and therapeutic outcomes.

Current studies indicate that excessive activation of ErbB tyrosine kinase family was associated with anoikis resistance in breast cancer or prostate cancers (24, 25), but the association of ErbB and anoikis has not yet been explored in HCC. In this study, we initially found that the ErbB signaling pathway stood out as the only upregulated one in the anoikis^high^ group among all bulk RNA-seq cohort. Then, the subgroup of anoikis^high^&ErbB^high^ patients had the worst prognosis compared to the other three subgroups, in which the NOTCH signaling pathway was enriched using bulk- and single-cell RNA sequencing (scRNA-seq). Through the leave-one-out cross-validation (LOOCV) framework, 167 machine learning procedures were constructed to predict the prognosis of HCC patients based on the Anoikis-ErbB related genes, and the LASSO + RSF model was selected for the optimal Anoikis-ErbB Related Signature (AERS), achieved the highest C-index, and demonstrated superior predictive power over 72 published predicting models. Additionally, AERS was shown to predict the response of patients with HCC to immune-targeted therapies.

## Material and methods

### Data collection

The gene expression profiles and clinical data were retrospectively obtained from the Cancer Genome Atlas (TCGA, https://cancergenome.nih.gov/), including 424 samples (involving 374 tumor tissues and 50 normal tissues), along with 240 tumor samples from the International Cancer Genome Construction (ICGC, https://dcc.icgc.org/projects/LIRI-JP). Moreover, GSE144269, GSE14520, and GSE116174 were downloaded from the Gene Expression Omnibus (GEO) databases (https://www.ncbi.nlm.nih.gov/geo/). The “ComBat” method within the R package “sva” was utilized to mitigate batch effects across the TCGA, ICGC, and GEO datasets. Furthermore, the GSE109211 cohort, consisting of 67 patients with HCC receiving sorafenib, was included to assess the efficacy of sorafenib response. The patients receiving anti-PD-1 treatment from the GSE91061 cohort (comprising 109 patients with melanoma), the phs000452 cohort (comprising 153 patients with melanoma who received anti-PD-1 treatment), and the Braun2020 cohort (comprising 311 patients with renal cell carcinoma) were obtained from TIGER database (http://tiger.canceromics.org/#/). Baseline characteristics of these cohorts are detailed in **Supplementary Table S1**.

### Clinical specimens

A total of 64 frozen HCC samples and 26 paracancer samples were collected from Mengchao Hepatobiliary Hospital (MCHH) between December 2015 and December 2018. Among these samples, 10 HCC samples had received sorafenib treatment for postoperative recurrence, and the response to sorafenib was assessed by two clinical experts. This study was ethically approved by the Ethics Committees of MCHH, with all patients providing written informed consent. The baseline characteristics of the MCHH cohort can be found in **Supplementary Table S2**.

### Analysis of pathway activity, function, immune infiltration, and drug sensitivity

The gene sets of anoikis and KEGG were extracted from the Gene Set Enrichment Analysis (GSEA) website (https://www.gsea-msigdb.org/GSEA/index.jsp, **Supplementary Table S3**). Then, single-sample GSEA (ssGSEA) and Gene Set Variation Analysis (GSVA) were employed using the R package “GSVA” to assess pathway activity. GSEA software (V4.3.3) was used for GSEA to identify the differentially regulated pathways. Moreover, the tumor immune microenvironment (TiME) was evaluated by the “EPIC” method using the R package “IBOR”. Additionally, several tools were applied to assess the predictive value of molecularly targeted drugs (MTDs) and immune checkpoint inhibitor (ICI) benefits. The half-maximal inhibitory concentration (IC_50_) of MTDs was calculated by the R package “pRRophetic”. The immunophenoscore (IPS) of patients with HCC was sourced from the Cancer Immunome Atlas (TCIA, https://tcia.at/home).

### Development and evaluation of a prognostic Anoikis-ErbB Related Signature via the machine learning-based integrative procedure

Differentially expressed genes (DEGs) were identified with the R package “limma”, and univariate Cox regression analysis was performed using the R package “survival” to screen for prognostic genes. A total of 167 types of machine learning integrations had been derived from 10 different machine learning algorithms, namely, Lasso, RSF, survival-SVM, SuperPC, plsRcox, GBM, Enet, Ridge, stepwise Cox, and CoxBoost. We determine the optimal hyperparameters for each machine learning model by utilizing the respective R package associated with each machine learning algorithm (**Supplementary Table S4**). The C-index, as calculated by Harrell, was utilized across all validation datasets to ascertain the optimal model.

Patients in each bulk RNA-seq cohort were stratified into AERS^low^ and AERS^high^ groups based on the median of AERS. The prognostic value of AERS was confirmed through analysis of survival differences using Kaplan–Meier (K-M) curves, time-dependent receiver operating characteristic (ROC) curves, and univariate and multivariate Cox regression analyses. These analyses were conducted using the R packages “survmine”, “survivalROC”, and “survival” respectively.

### The scRNA-seq data quality control and analysis

Initially, the scRNA-seq cohort GSE149614 including 10 tumor samples was extracted from GEO databases. Data process and quality control were conducted by the R package “Seurat”. A total of 16,709 cells were filtered out based on specific criteria, including the requirement that each gene be expressed in a minimum of three cells, each cell express at least 250 genes, the gene count per cell falls within a range of 100 to 5,000, and mitochondrial gene expression be maintained below 25%. Furthermore, the filter condition of unique molecular identifier (UMI) counts ranging from 100 to 50,000 was considered. Additionally, the scRNA-seq data were normalized and batch effects were removed using the R package “harmony”. Following this, principal component analysis (PCA) was conducted to reduce the dimensionality of cells, with unsupervised cell clusters generated under the conditions of dim = 20 and resolution = 0.2. Moreover, classical immune cell markers were employed for annotating subpopulations, with data sourced from the CellMarker 2.0 database (http://biocc.hrbmu.edu.cn/CellMarker/). In addition, the identification of phenotype-guided single-cell subpopulations was also carried out using the R package “scissor”. To evaluate the functions of pathway, analysis by R package GSVA was performed. In order to deduce the cellular development trajectory, we performed pseudotime analyses and trajectory construction in the R package “monocle”. After that, we delved deeper into cell–cell communication by employing the R package “CommPath” and visualizing signaling pathway networks within clusters through heatmap.

### Quantitative real-time polymerase chain reaction

Previous studies served as the basis for conducting RNA extraction and reverse transcription procedures (26). Target gene expression was normalized to β-actin, with all quantitative real-time polymerase chain reaction (qRT-PCR) analyses conducted in triplicate. Primer sequences for the five target genes and β-actin are listed in **Supplementary Table S5**.

### Statistical analyses

This study utilized R-4.1.2 for statistical analyses. Chi-square test was used to evaluate differences in proportions. The normality of variables was assessed using the Shapiro–Wilk test. Student’s *t*-test was employed to assess differences between two groups with normally distributed variables, while the Wilcoxon test was used for variables that were not normally distributed. Parametric analysis of variance (ANOVA) tests were conducted for multiple group comparisons, while nonparametric Kruskal–Wallis tests were utilized. Spearman correlation and distance correlation analyses were performed based on correlation coefficients. *p* < 0.05 indicates statistical significance.

## Results

### The clinical significance and potential mechanism of anoikis and ErbB levels

**Figure 1:**
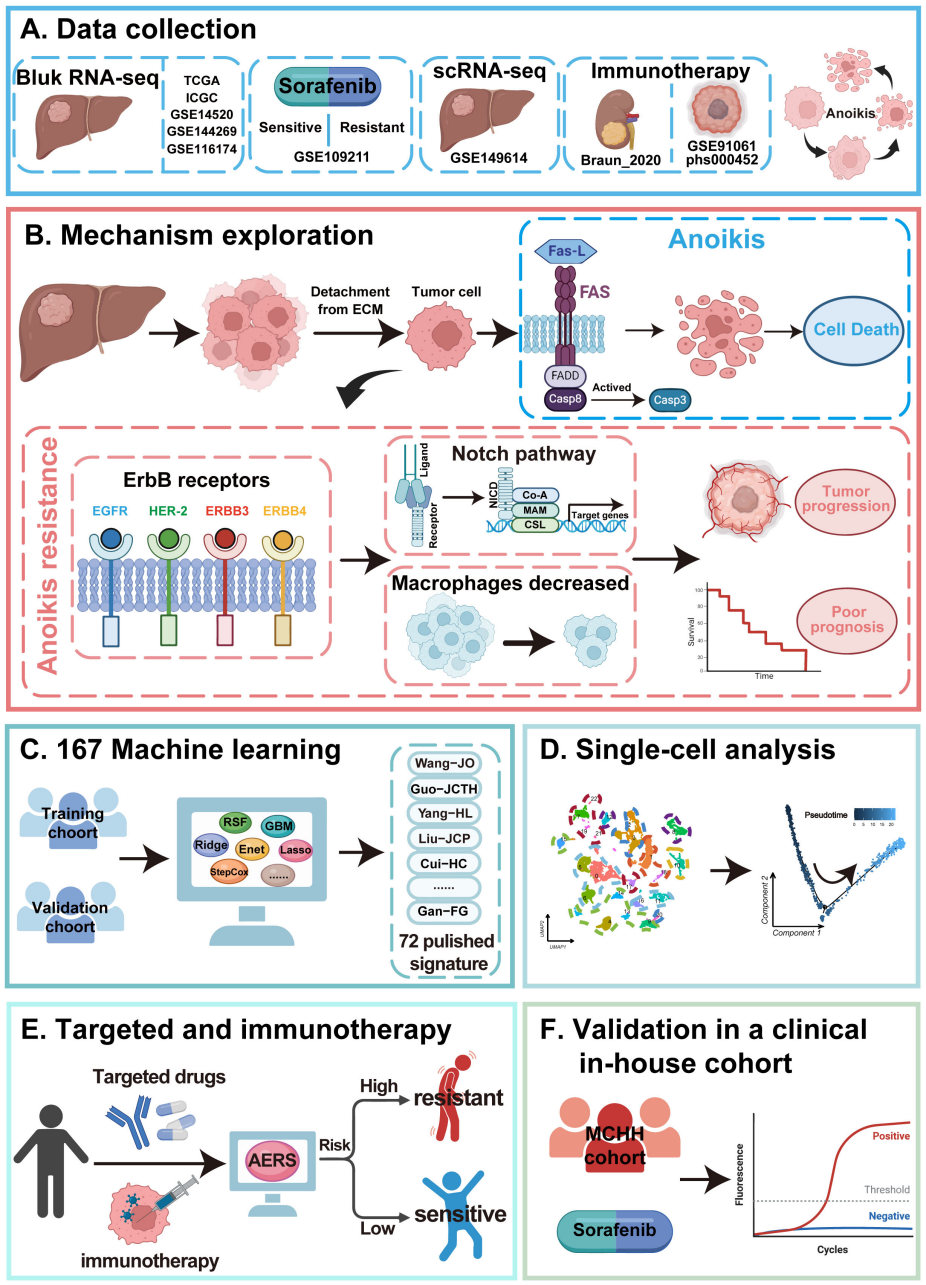
Flowchart of the present study.

**Figure 2:**
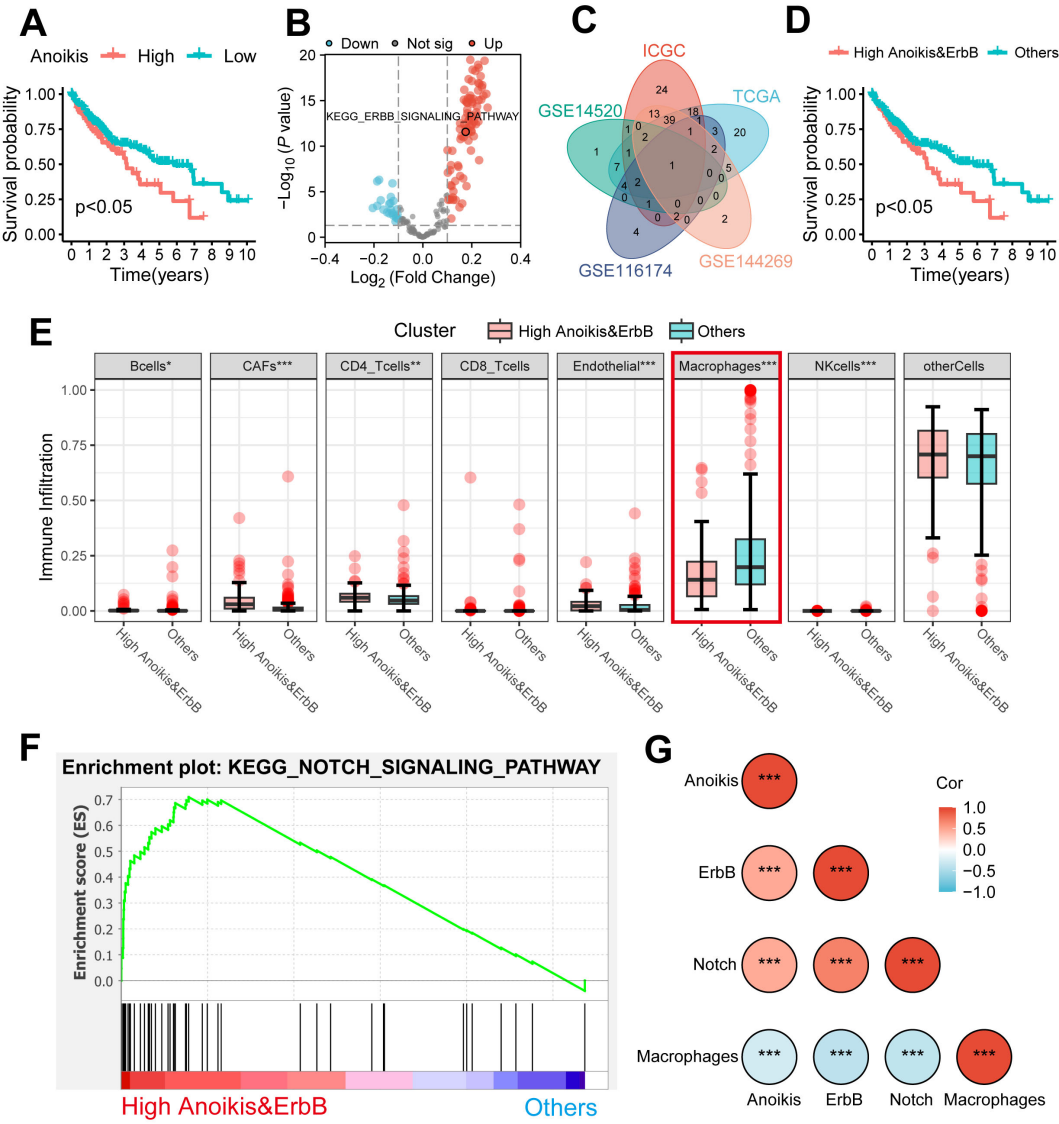
The clinical significance and potential mechanism of anoikis and ErbB levels. **(A)** Kaplan–Meier analysis of OS stratified based on ssGSEA scores of anoikis level. **(B)** Volcano plot showed the GSVA of differential pathways stratified based on anoikis level. **(C)** The ErbB pathway was identified via a Venn diagram. **(D)** Kaplan–Meier analysis of OS stratified based on the combination of anoikis and ErbB pathway level. **(E)** Differential immune cell infiltration analysis between the anoikis^high^&ErbB^high^ group and others. **(F)** The NOTCH pathway was identified between the anoikis^high^&ErbB^high^ group and others via GSEA. **(G)** Heatmap shows the correlations among anoikis level, ErbB pathway level, NOTCH pathway level, and macrophage infiltration. OS, overall survival; ssGSEA, single sample Gene Set Enrichment Analysis; GSVA, Gene Set Variation Analysis; GSEA, Gene Set Enrichment Analysis. *p < 0.05; ** *p* < 0.01; ***p < 0.001.

**Figure 1** illustrates the study workflow. In the TCGA cohort, ssGSEA was applied to assess anoikis levels in patients with HCC, revealing a significant association between elevated anoikis levels and worse prognosis (**Figure 2A**, *p* < 0.05). To further explore the potential relationship between anoikis levels and HCC prognosis, GSVA of the KEGG pathway was conducted. Interestingly, ErbB pathway stood out as the only one that was upregulated in all bulk RNA-seq cohorts, including TCGA, ICGC, GSE14520, GSE144269, and GSE116174 cohorts (**Figures 2B, C**, as detailed in **Supplementary Table S6**). Similarly, high ErbB pathway activity also led to worse prognosis in patients with HCC (**Supplementary Figure S1A**, *p* < 0.05). Prognostic analysis of both pathways demonstrated that patients in the anoikis^high^&ErbB^high^ group had the most unfavorable prognosis compared to other subgroups (**Figure 2D**; **Supplementary Figure S1B**, all *p* < 0.05). Based on the above findings, we hypothesize that the anoikis and ErbB pathways engage in the same biological processes to influence HCC prognosis. Immune infiltration analysis indicated that macrophage was significantly downregulated in the anoikis^high^&ErbB^high^ group (**Figure 2E**, *p* < 0.001), alongside the upregulation of the NOTCH pathway (**Figure 2F**). Correlation analysis indicated a positive relationship between anoikis and ErbB levels with NOTCH activity, and a significant negative correlation with macrophage infiltration (**Figure 2G**, as detailed in **Supplementary Figures S2A-F**). Similar patterns were observed in the meta-cohort (**Supplementary Figures S3A-E**).

**Figure 3:**
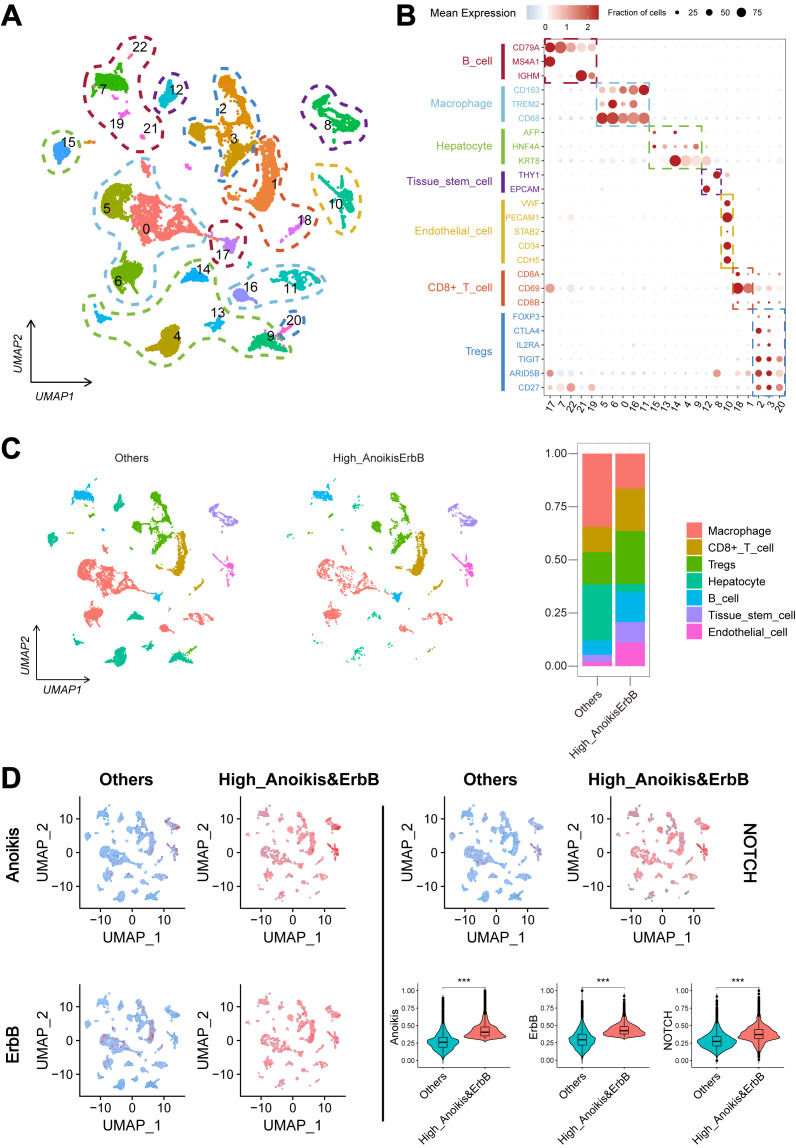
Landscape and potential mechanism of HCC via scRNA-seq. **(A)** The UMAP plot of 22 cell clusters from the multicellular ecosystem of 10 HCC patients. **(B)** Dotplot showed the percentage of expressed cells and average expression levels of canonical marker genes of major cell types in 22 cell clusters. **(C)** UMAP plot and bar plots indicate the landscape and proportion of major cell lineages between anoikis^high^&ErbB^high^ and the other cells. **(D)** UMAP plot and violin plot showed the distribution of anoikis level, ErbB pathway level, and NOTCH pathway level between anoikis^high^&ErbB^high^ and the other cells. HCC, hepatocellular carcinoma; scRNA-seq, single-cell RNA sequencing; UMAP, Uniform Manifold Approximation and Projection. ***p < 0.001.

Further validation was performed using the scRNA-seq cohort (GSE149614), where 22 infiltration clusters were identified via PCA and visualized through UMAP plots (**Figure 3A**). Then, the annotation and division of the main infiltration clusters were performed with typical marker genes for Tregs, CD8+ T cells, endothelial cells, tissue stem cells, hepatocytes, macrophages, and B cells (**Figure 3B**). GSEA identified cells with anoikis^high^&ErbB^high^ characteristics, revealing a significant reduction in macrophage levels compared to other cells (**Figure 3C**) while showing elevated anoikis, ErbB, and NOTCH activity (**Figure 3D**). These results suggest that anoikis may affect HCC prognosis by enhancing NOTCH pathway activity and reducing macrophage infiltration through ErbB receptor signaling.

### Construction and evaluation of AERS based on hub genes of anoikis and ErbB

**Figure 4:**
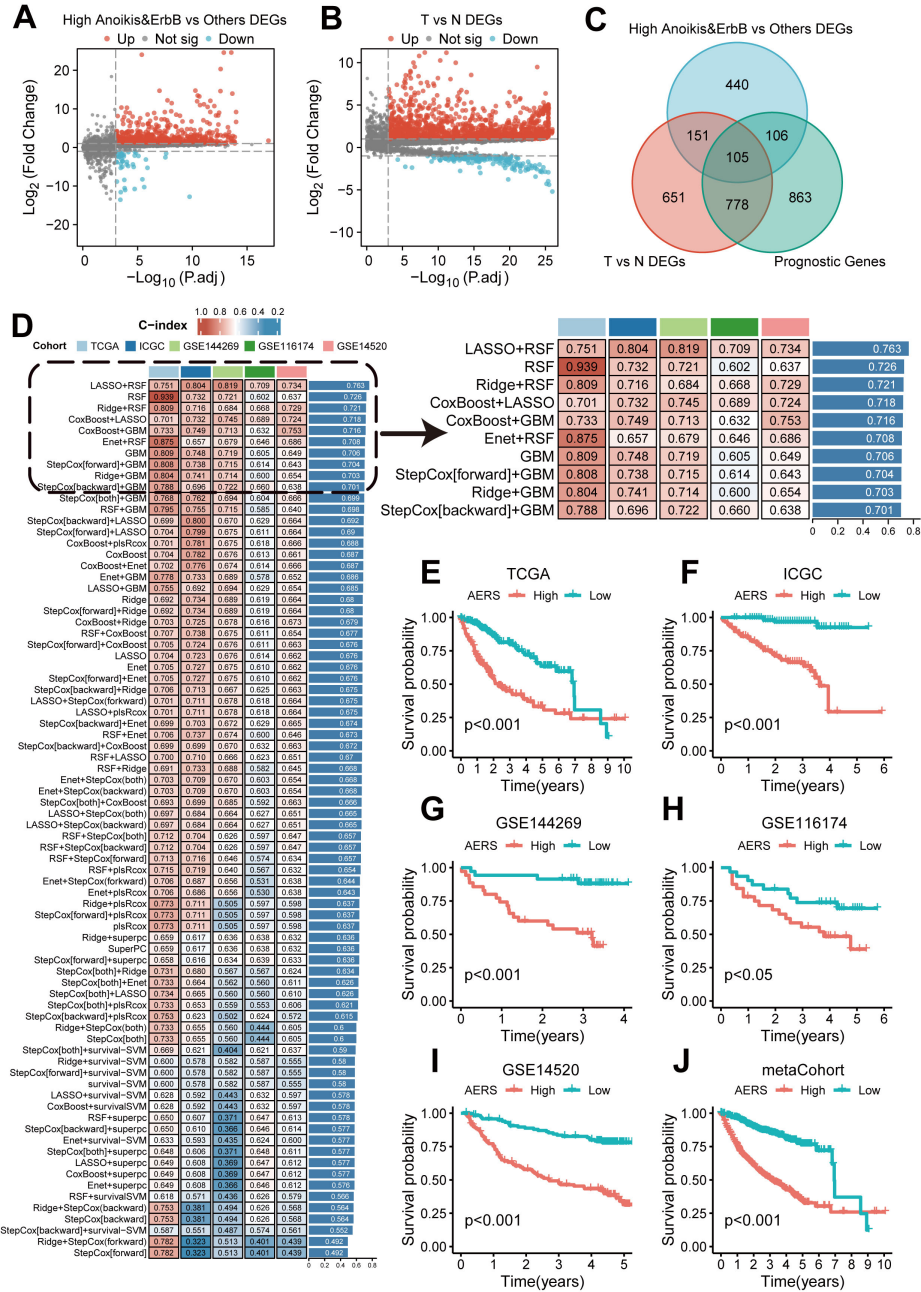
Construction of AERS based on anoikis and ErbB pathway. **(A)** Differential expression analysis results revealed that significant genes were upregulated and downregulated in the anoikis^high^&ErbB^high^ group with the TCGA cohort. **(B)** Differential expression analysis results revealed that significant genes were upregulated and downregulated in tumor samples with the TCGA cohort. **(C)** The 105 candidate genes were identified via a Venn diagram in the TCGA cohort. **(D)** A total of 167 prediction models via the LOOCV framework and the C-index of each model across all cohorts. **(E–J)** Kaplan–Meier curves of OS with different AERS groups in TCGA, ICGC, GSE144269, GSE116174, GSE14520, and mate-cohort. AERS, Anoikis& ErbB-related signature; TCGA, The Cancer Genome Atlas; LOOCV, leave- one-out cross-validation; OS, overall survival; ICGC, International Cancer Genome Construction.

A total of 802 DEGs were identified between the anoikis^high^&ErbB^high^ group and other patients (**Figure 4A**, as detailed in **Supplementary Table S7**), while 1,685 DEGs were identified between tumor and adjacent non-tumor tissues (**Figure 4B**, as detailed in **Supplementary Table S8**). In addition, 1,852 genes were identified as prognostic genes (**Supplementary Table S9**). Venn analysis revealed 105 candidate genes (**Figure 4C**). Utilizing the LOOCV framework, 167 machine learning-based procedures were employed to construct the AERS for predicting HCC prognosis in the TCGA cohort, and a combination of LASSO and RSF algorithms yielding the highest mean C-index of 0.763 (**Figure 4D**).

AERS was constructed based on five key genes (CCT2, MARCKSL1, SLC2A1, ECT2, and CDK4), as demonstrated by K-M curves and univariate Cox regression analysis in **Supplementary Figures S4A-L** (all *p* < 0.001). Patients were stratified into two groups according to the median AERS score of AERS, and K-M curves show that the AERS^high^ group had a significantly worse prognosis across all cohorts (**Figures 4E–J**, all *p* < 0.05).

**Figure 5:**
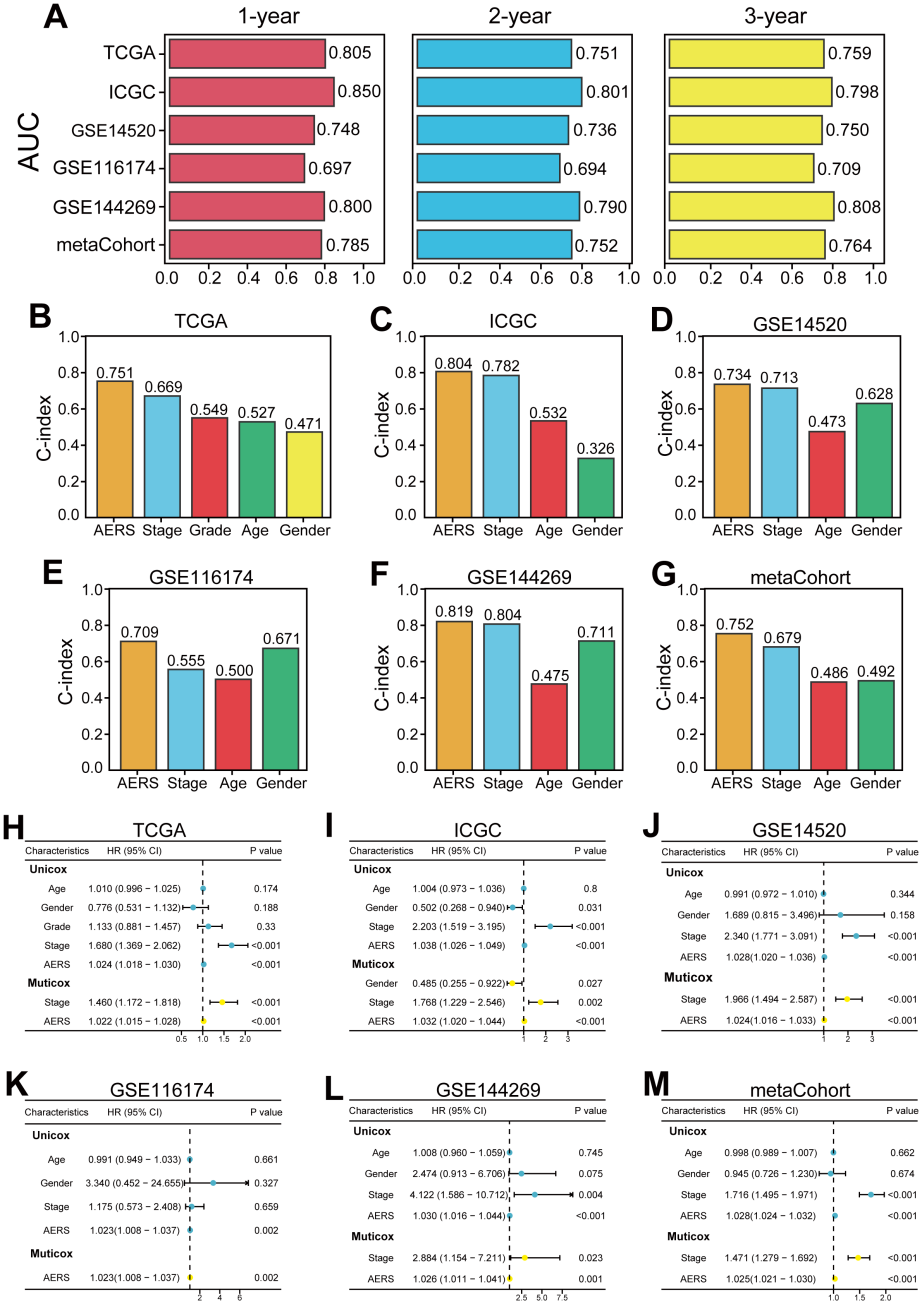
Evaluation of the AERS in multiple cohorts. **(A)** Time-dependent ROC analysis of AERS for predicting OS at 1, 2, and 3 years in the cohorts of TCGA, ICGC, GSE144269, GSE116174, GSE14520, and mate-cohort. **(B–G)** C-index of AERS compared with other clinical characteristics in predicting prognosis. **(H–M)** Univariate and multivariate Cox regression analysis of AERS and other clinical characteristics. ROC, receiver operating characteristic; AERS, Anoikis& ErbB-related signature; OS, overall survival; TCGA, The Cancer Genome Atlas; ICGC, International Cancer Genome Construction.

The area under the curve (AUC) at 1 year for the TCGA, ICGC, GSE14520, GSE116174, and GSE144269 cohorts and meta-cohort were 0.805, 0.850, 0.748, 0.697, 0.800, and 0.785, respectively. At 2 years, AUC values were 0.751, 0.801, 0.736, 0.694, 0.790, and 0.752; at 3 years, they were 0.759, 0.798, 0.750, 0.709, 0.808, and 0.764, respectively (**Figure 5A**). The C-index values for these cohorts were 0.751, 0.804, 0.734, 0.709, 0.819, and 0.752, indicating superior prognostic power compared to clinical features such as age, gender, stage, and grade (**Figures 5B-G**). Multivariate Cox regression analysis confirmed AERS as an independent risk factor for overall survival (OS) in all cohorts (**Figures 5H–M**, all *p* < 0.05).

By reviewing previously reported prognostic signatures for HCC, 72 relevant signatures covering various biological processes—including stemness, autophagy, ferroptosis, epithelial–mesenchymal transition, and immune response—were identified. Univariate Cox regression analysis demonstrated that AERS was the only prognostic signature across all cohorts (**Supplementary Figure S5A**, all *p* < 0.05). Furthermore, compared to the 72 signatures, AERS had the highest C-index and demonstrated unmatched prognostic predictive capability in all cohorts (**Supplementary Figures S5B-G**).

### The potential mechanism for poor prognosis of the AERS^high^ subgroup via scRNA-seq

**Figure 6:**
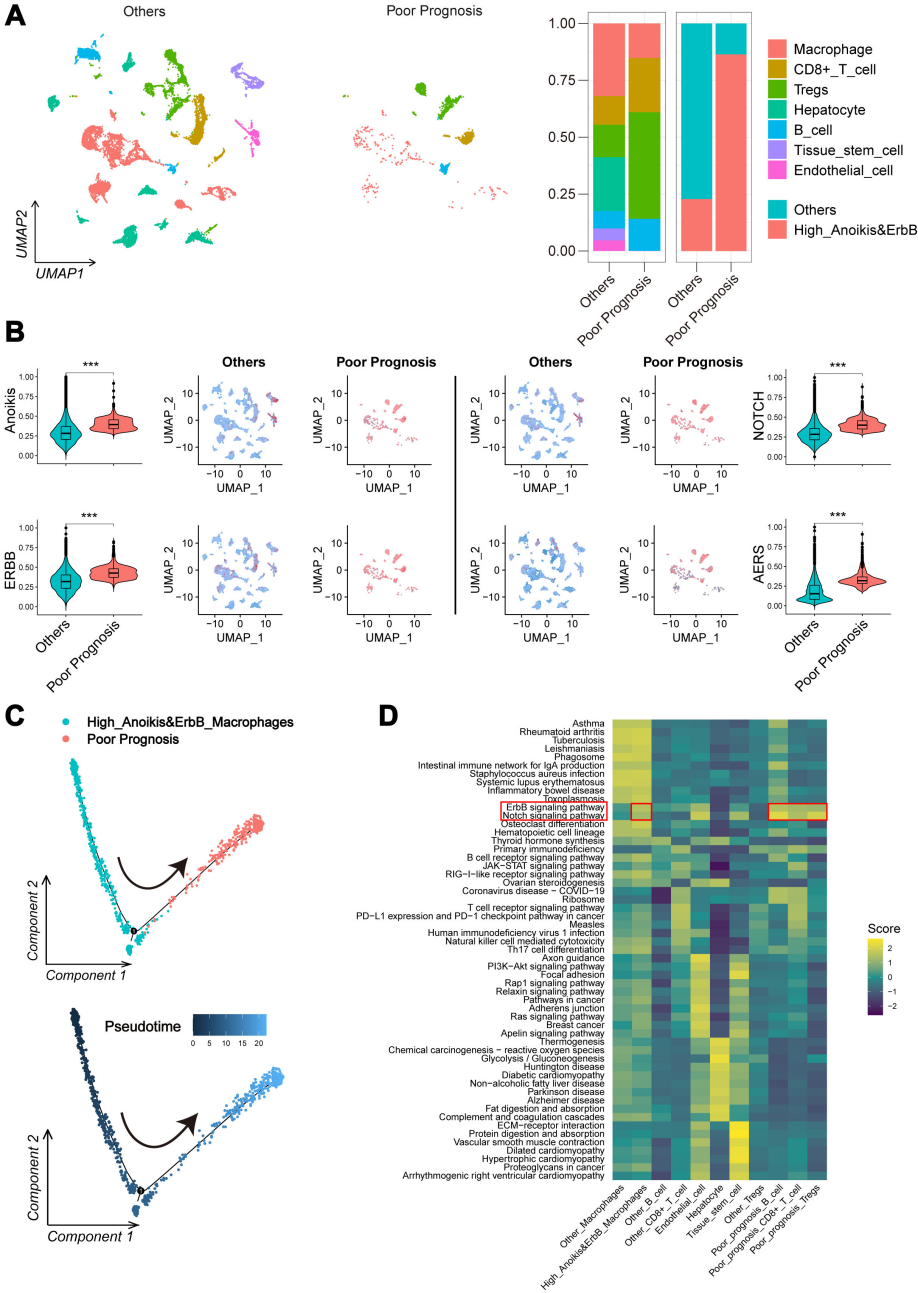
Potential mechanism of AERS in HCC progression. **(A)** UMAP plot and bar plots indicates the landscape and proportion of major cell types between the poor prognosis group and the other cells. **(B)** UMAP plot and violin plot shows the distribution of anoikis level, ErbB pathway level, NOTCH pathway level, and AERS between the poor prognosis group and the other cells. **(C)** Pseudotime analysis for the anoikis^high^&ErbB^high^ macrophages with poor prognosis. **(D)** Heatmap indicates the activation of major pathways in cell subpopulation with poor prognosis. AERS, Anoikis& ErbB-related signature; HCC, hepatocellular carcinoma; scRNA-seq, single-cell RNA sequencing; UMAP, Uniform Manifold Approximation and Projection. ***p < 0.001.

Further validation of AERS-related biological processes was performed through scRNA-seq cohort analysis. Cells with poor prognostic characteristics were identified using the “scissor” algorithm. Consistent with findings from the anoikis^high^&ErbB^high^ group, a significant reduction in macrophage abundance and landscape was observed compared to other cell types (**Figure 6A**), while anoikis, ErbB, NOTCH, and AERS levels were notably elevated (**Figure 6B**). Additionally, pseudotime analysis revealed a clear trajectory indicating that anoikis^high^&ErbB^high^ cells tend to transition into poor prognosis cells (**Figure 6C**). Cellular communication analysis, conducted via the “CommPath” method, further explored ligand–receptor interaction counts and intensity among cell subpopulations (**Supplementary Figures S6A, B**). GSEA identified ErbB and NOTCH signaling pathways as activated in both anoikis^high^&ErbB^high^ cells and poor prognosis cells (**Figure 6D**). These results collectively suggest that elevated AERS may contribute to poor prognosis by activating ErbB and NOTCH signaling and reducing macrophage infiltration.

### Molecular targeted drugs and immune checkpoint inhibitors related to AERS

**Figure 7:**
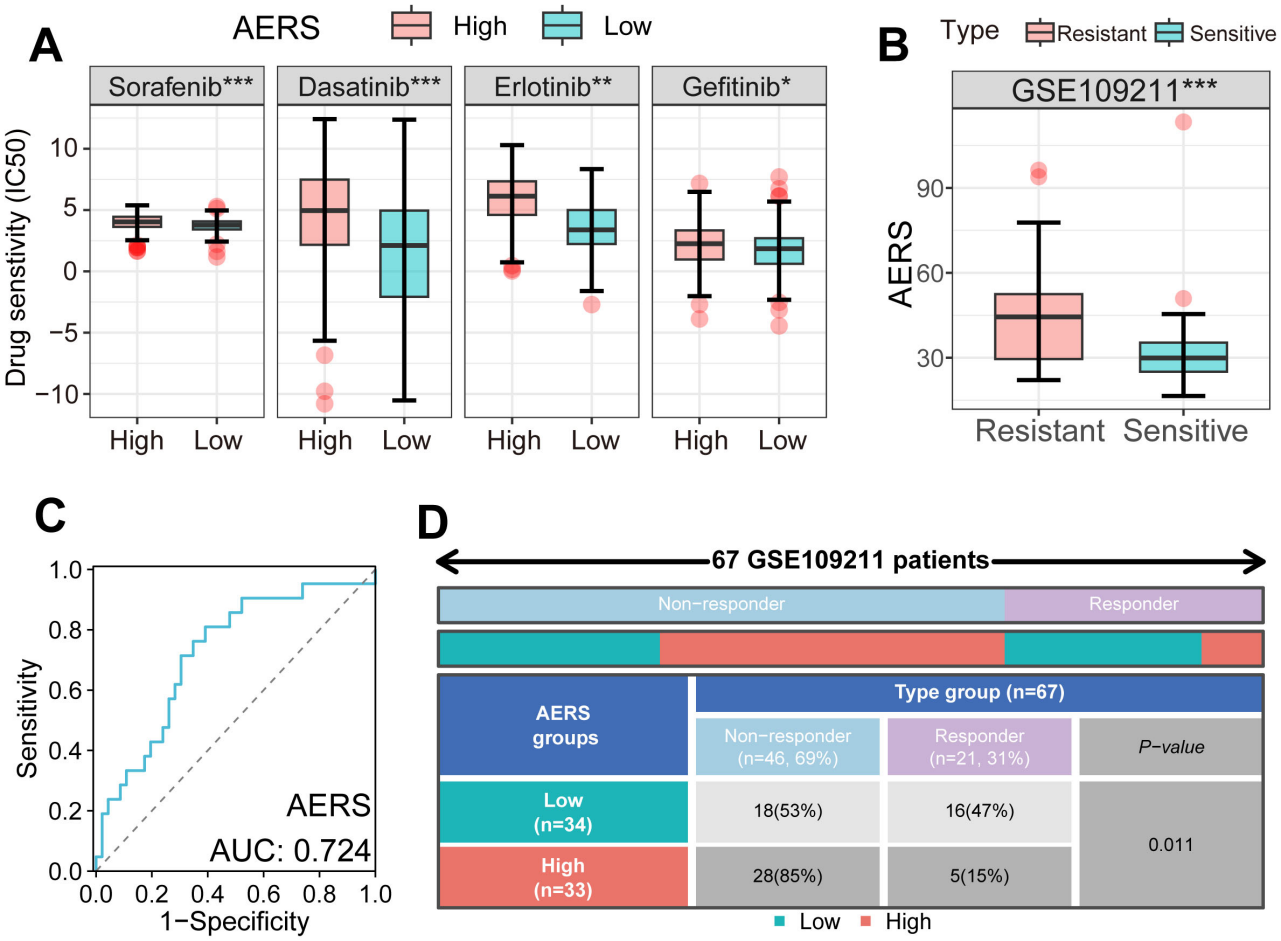
Application of AERS in MTDs. **(A)** Drug sensitivity analysis showed an IC_50_ of four MTDs in different AERS groups in the TCGA cohort. **(B)** The distribution of the differential AERS between response and non-response groups in the GSE109211 cohort. **(C)** ROC curve of AERS to predict sorafenib response in the GSE109211 cohort. **(D)** Fourfold table between AERS and sorafenib response in the GSE109211 cohort. MTDs, molecular targeted drugs; AERS, Anoikis& ErbB-related signature; HCC, hepatocellular carcinoma; IC_50_, half-maximal inhibitory concentration; ROC, receiver operating characteristic. *p < 0.05; **p < 0.01; ***p < 0.001.

Using the “pRRophetic” algorithm to assess drug sensitivity to MTDs, the IC_50_ of sorafenib, erlotinib, dasatinib, and gefitinib was found to be significantly higher in the AERS^high^ group within the TCGA cohort (**Figure 7A**). In the GSE109211 cohort, sorafenib-resistant patients exhibited notably higher AERS scores (**Figure 7B**), with an AUC of AERS to predict sorafenib response (**Figure 7C**). Additionally, the sorafenib response rate to sorafenib was significantly lower in the AERS^high^ group compared to the AERS^low^ group (**Figure 7D**, *p* < 0.05). Interestingly, ErbB and NOTCH pathway activation was also observed in sorafenib-resistant cells (**Supplementary Figures S7A–D**, **S8A, B**), indicating that AERS may predict responses not only to sorafenib but also to other MTDs.

**Figure 8:**
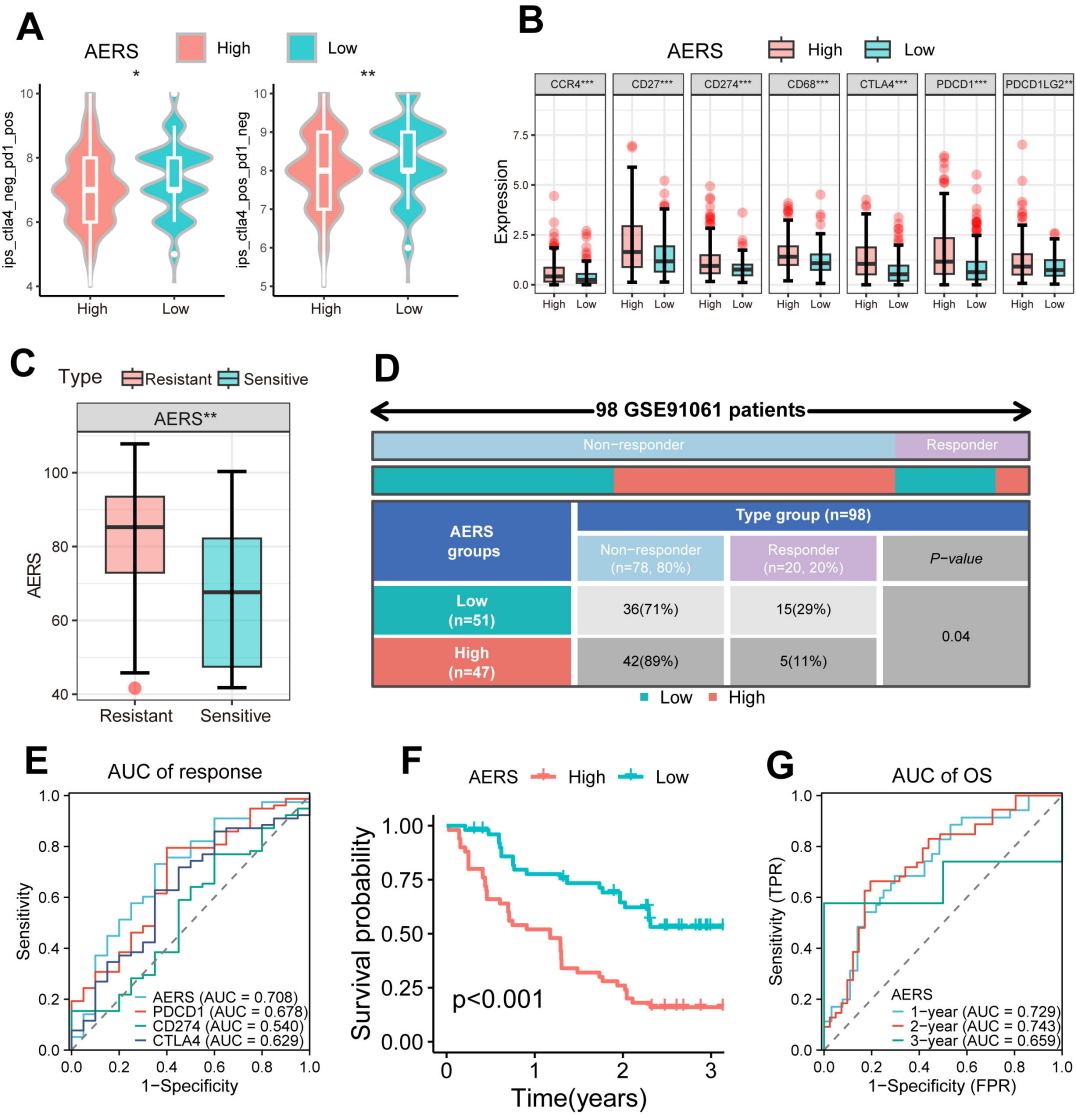
Application of AERS in ICIs. **(A)** Violin plot shows the distribution of IPS score between different AERS groups in the TCGA cohort. **(B)** The distribution of the differential immune checkpoint molecule expression between different AERS groups in the TCGA cohort. **(C)** The distribution of the differential AERS between sensitive and resistant groups in the GSE91061 cohort. **(D)** Fourfold table between AERS and ICI response in the GSE91061 cohort. **(E)** ROC curve of AERS to predict immunotherapy response in the GSE91061 cohort. **(F)** Kaplan–Meier curves of OS with different AERS groups in the GSE91061 cohort. **(G)** Time-dependent ROC analysis of AERS for predicting OS at 1, 2, and 3 years in the GSE91061 cohort. ICIs, immune checkpoint inhibitors; AERS, Anoikis& ErbB-related signature; TCGA, The Cancer Genome Atlas; ROC, receiver operating characteristic. *p < 0.05; **p < 0.01; ***p < 0.001.

The IMbrave150 trial introduced the combination of MTDs and ICIs; therefore, we tried to explore the predictive value of AERS in patients with HCC receiving ICIs (4). IPS, commonly used to predict ICI response, was significantly decreased in the AERS^high^ group (**Figure 8A**, all *p* < 0.05). Additionally, immune checkpoint molecules (e.g., CCR4, CD27, CD274, CD68, CTLA4, PDCD1, and PDCD1LG2) were significantly upregulated in the AERS^high^ group (**Figure 8B**, all *p* < 0.01), suggesting potential impairment of the anti-tumor immune response. Analysis of the GSE91061 cohort, consisting of 109 patients with melanoma receiving anti-PD-1, revealed higher AERS scores in the resistant group (**Figure 8C**, *p* < 0.01), and the ICI response rate was significantly lower in the AERS^high^ group compared to the AERS^low^ group (**Figure 8D**). The AUC for AERS in predicting ICI response was 0.708, outperforming common immune checkpoints like PDCD1, CD274, and CTLA4 (**Figure 8E**). Patients in the AERS^high^ group also had worse survival outcomes (**Figure 8F**, *p* < 0.001), with 1-, 2-, and 3-year AUCs of 0.729, 0.743, and 0.659, respectively (**Figure 8G**). Similar results were observed in the phs000452 cohort (including 153 patients with melanoma) and the Braun2020 cohort (including 311 patients with renal cell carcinoma), both treated with anti-PD-1 (**Supplementary Figures S9A-J**).

### The validation of AERS in a clinical in-house cohort

**Figure 9:**
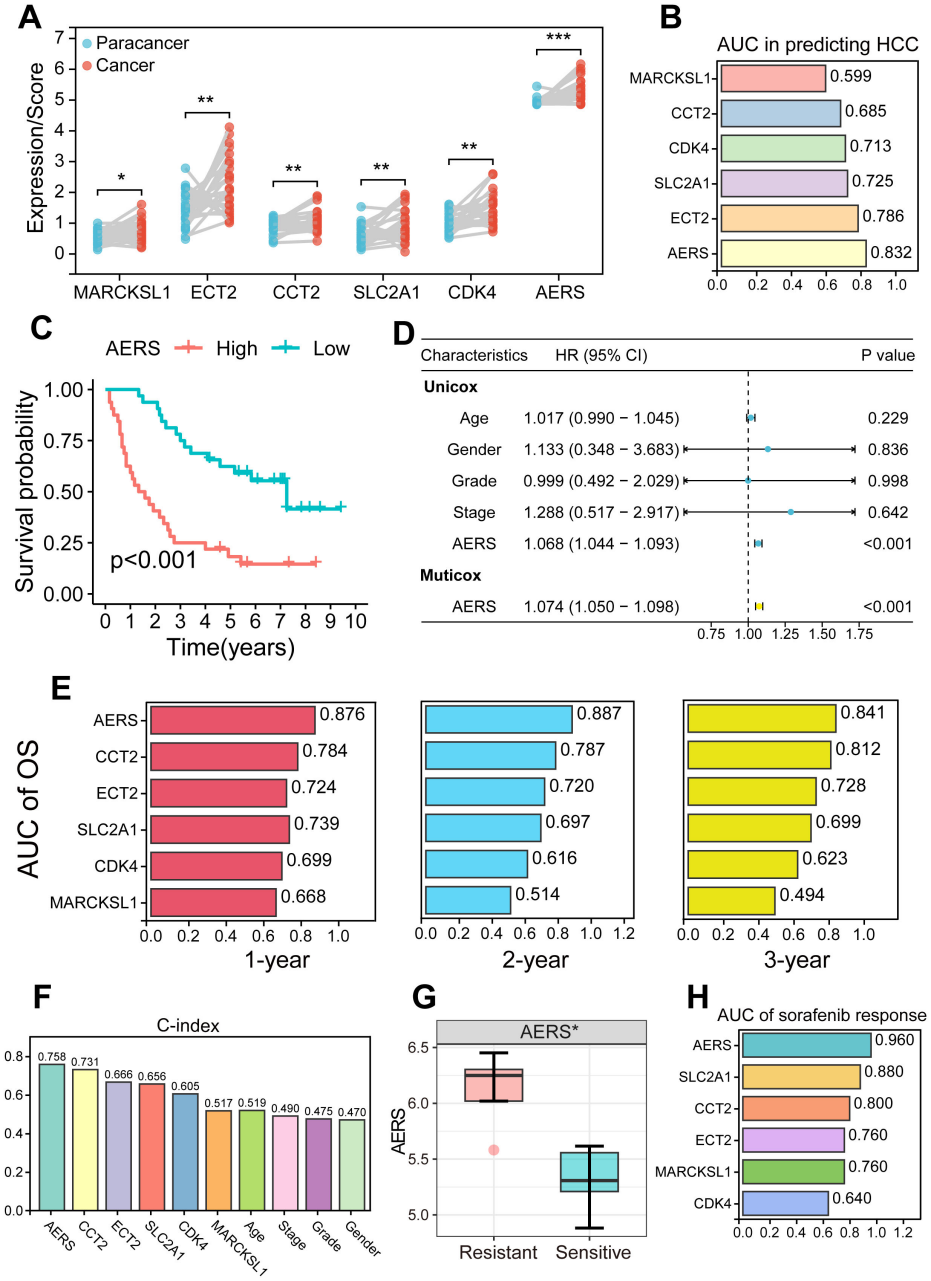
Validation of AERS by an in-house cohort. **(A)** Paired plot shows the distribution of signature genes (CCT2, MARCKSL1, SLC2A1, ECT2, and CDK4) expression and AERS scores between HCC and paracancer samples. **(B)** ROC analysis of signature genes and AERS to predict HCC. **(C)** Kaplan–Meier curves of OS according to AERS. **(D)** Univariate and multivariate Cox regression analysis of AERS and other clinical characteristics. **(E)** Time-dependent ROC analysis for predicting OS at 1, 2, and 3 years according to signature genes expression and AERS in the clinical in-house cohort. **(F)** C-index of AERS compared with signature genes and other clinical characteristics in predicting prognosis. **(G)** The distribution of the differential AERS between sorafenib response and non-response groups. **(H)** ROC analysis of signature genes and AERS to predict sorafenib response. AERS, Anoikis& ErbB-related signature; HCC, hepatocellular carcinoma; ROC, receiver operating characteristic; OS, overall survival. *p < 0.05; **p < 0.01; ***p < 0.001.

To further validate the clinical applicability and predictive power of AERS, qRT-PCR was performed to assess the expression of the five signature genes in 64 HCC samples and 26 paracancerous samples from MCHH, including 10 patients with HCC who experienced postoperative recurrence and received sorafenib treatment. The expression levels of the five genes and AERS were significantly elevated in tumor tissues (**Figure 9A**, all *p* < 0.05), with AERS showing the highest AUC for distinguishing tumor tissue at 0.832, outperforming individual signature genes (**Figure 9B**).

Survival analysis in the in-house cohort further confirmed the prognostic value of AERS. Patients in the AERS^high^ group exhibited significantly worse prognosis (**Figure 9C**, *p* < 0.001), and multivariate Cox regression analysis identified AERS as an independent risk factor for OS (**Figure 9D**, *p* < 0.001). K-M analysis of the five signature genes is shown in **Supplementary Figures S10A–E**, where all genes, except MARCKSL1, were significantly associated with OS in univariate Cox regression analysis (**Supplementary Figure S10F**, *p* < 0.001). AERS demonstrated the highest AUC at 1, 2, and 3 years (0.876, 0.887, and 0.841, respectively) when compared to the five signature genes (**Figure 9E**). Additionally, AERS achieved a C-index of 0.758, which indicated a most superior prognostic predictive power compared to both the signature genes and other clinical characteristics, such as age, gender, stage, and grade (**Figure 9F**).

Moreover, the patients who were resistant to sorafenib treatment had a significantly higher AERS (**Figure 9G**, *p* < 0.05), with an AUC of 0.960 for predicting sorafenib response (**Figure 9H**). Notably, aside from CDK4 in the gender subgroup, no significant differences were observed in the expression levels of AERS and the signature genes across various clinicopathologic characteristics (**Supplementary Figures S11A–D**, all P>0.05).

## Discussion

Prognostication remains a critical concern in HCC (27). Previously published studies primarily concentrate on a single gene or gene set, raising questions about their reproducibility and generalizability across diverse patient populations and clinical settings (28, 29). In this investigation, we integrated two prominent gene sets of anoikis and ErbB to develop 167 prognostic models for HCC using machine learning techniques. The LASSO + RSF model emerged as the optimal AERS, demonstrating superior predictive capability compared to existing prognostic scores. Further analyses identified a correlation between elevated AERS, activated NOTCH signaling pathways, and decreased macrophage infiltration, as observed through both bulk and scRNA sequencing. These biological changes are associated with resistance to sorafenib and ICIs (30).

Anoikis and ErbB signaling pathways are well-documented for their roles in the progression and prognosis of HCC (14, 31, 32). Despite the establishment of numerous risk scores based on these pathways (31, 33), their interactions remain underexplored. In this study, ErbB signaling was the only upregulated one in the anoikis^high^ group among all bulk RNA-seq cohort, and elevated anoikis score or ErbB score was negatively correlated with the prognosis of HCC patients. Then, we found that patients with anoikis^high^&ErbB^high^ had the worst prognosis compared with other subgroups and were much more likely to resist to TKIs. Analyses of bulk and scRNA sequencing revealed that the NOTCH signaling pathway was activated in the anoikis^high^&ErbB^high^ subgroup, accompanied by decreased macrophage infiltration, both of which were reported to be associated with poor prognosis and therapeutic resistance in HCC (34).

Utilizing an LOOCV framework, 10 machine learning algorithms were applied to generate 167 model combinations. This integrative approach reduces dimensionality of variables, enhances stability, and mitigates overfitting, making it highly promising for clinical application. The principal innovation of this research lies in the creation of 167 prognostic models for HCC using machine learning, based on Anoikis-related and ErbB-related genes. Then, according to the mean ROC, we selected LASSO+RSF as the optimal AERS, the C-index of which was higher than the other clinicopathogical characteristics (age, gender, grading, and stage). Of note, the current AERS had better predicting power than the other 72 published predicting models across all the cohorts. The current AERS incorporated five genes, namely, MARCKSL1, CCT2, CDK4, SLC2A1, and ECT2. MARCKSL1 is a member of the MARCKS family of protein kinase C substrates, known for playing a role in actin cytoskeleton remodeling and cellular signaling (35). CCT2 is part of a group of proteins known as chaperonins, which are specifically involved in the complex process of folding actin and tubulin (36). CDK4 is a key protein in cell cycle regulation, which is critical for cellular proliferation (37). SLC2A1, also known as GLUT1, plays a crucial role in glucose uptake and metabolic processes (38). ECT2 has roles in controlling the cytoskeleton and is also studied for its involvement in tumor formation and progression (39). Notably, the current AERS and the incorporated five genes were also verified in an in-house cohort. This comprehensive integration of gene sets into machine learning models represents a significant leap in HCC prognostication, underscoring its potential utility in guiding treatment strategies.

Sorafenib remains a cornerstone of systemic therapy for advanced HCC, yet resistance emerges in a significant portion of patients, curbing its long-term efficacy (40). The mechanisms of sorafenib resistance are multifactorial and include cellular adaptations, tumor microenvironment interactions, and genetic and epigenetic alterations (41–43). In this study, we noted that the AERS^high^ subgroup was much more likely to resist to sorafenib than the AERS^low^ subgroup based on the current AERS score. More importantly, bulk and scRNA sequencing analysis revealed that the activated NOTCH signaling pathway and decreased macrophage infiltration might be the answer for sorafenib resistance in this subgroup. In the context of sorafenib resistance, the NOTCH signaling pathway has been implicated in the promotion of cancer stem cell, regulation of tumor microenvironment, and induction of epithelial-to-mesenchymal transition (EMT) (44). Evidence revealed that macrophages also played an important role in sorafenib resistance (30, 45). Consequently, targeting the NOTCH pathway and macrophage polarization may serve as viable strategies to mitigate resistance to sorafenib.

Emerging as a potent therapeutic option, ICIs have shown promise in treating advanced HCC, particularly in combination with targeted therapies (4, 5, 46). Application of PD-L1 expression, tumor mutational burden (TMB), and microsatellite instability (MSI) greatly improves patient selection and predicting responses to treatment (47, 48), but challenges remain in achieving consistent biomarker validation across diverse populations and cancer types, as well as in integrating complex biomarkers like immune cell infiltration and gene expression profiles into routine clinical practice to guide therapy decisions more effectively (49, 50). In our analysis, we found that the AERS^high^ group exhibited decreased IPS of PD1 and CTLA4, alongside increased expression of immune checkpoints PDCD1, CD274, CTLA4, and PDCD1LG2. These findings indicated that this population might be resistant to ICIs, which was confirmed by three independent validation cohorts receiving anti-PD1 treatment. Further analysis revealed that AERS exhibited better predicting power for ICI response than traditional biomarkers of PDCD1, CD274 and CTLA4. Collectively, AERS could also be a potential biomarker for patient selection and predicting response to ICIs.

Despite these findings, several limitations exist in the study. First, this research was based on retrospective cohorts and requires validation through large-scale, multi-center prospective trials. Second, although AERS displayed strong predictive capabilities to survival and immune-targeted therapy outcomes in HCC, it may not fully capture the complexity of HCC biology and treatment responses. Third, while AERS has been validated in transcriptomics using RT-qPCR, further proteomic analyses are necessary to substantiate these results. Finally, the mechanisms underlying immune-targeted therapy resistance driven by anoikis&ErbB need further validation *in vitro* and *in vivo*.

## Conclusion

In conclusion, this study enhances HCC prognostication by integrating the key genes identified by the anoikis&ErbB pathway into 167 machine learning-based models, with AERS (combination of the LASSO and RSF model) showing superior predictive accuracy over existing scores. The AERS^high^ subgroup, characterized by activated NOTCH signaling and decreased macrophage infiltration, demonstrated resistance to immune-targeted therapies. These findings highlight the potential of targeting specific biological pathways to overcome therapeutic resistance and improve treatment outcomes in HCC, offering a promising avenue for personalized medicine in this challenging disease landscape.

## Data Availability

The datasets presented in this study can be found in online repositories. The names of the repository/repositories and accession number(s) can be found in the article/**Supplementary Material**.

## Supplementary material

Supplementary Figure 1 Kaplan-Meier analysis of OS stratified based on ssGSEA scores of ErbB level **(A)**, as well as anoikis&ErbB levels **(B)**.

Supplementary Figure 2
**(A-F)** Scatter plot shows the correlations among anoikis level, ErbB pathway level, NOTCH pathway level and macrophage infiltration.

Supplementary Figure 3 The clinical significance and potential mechanism exploration of anoikis levels in meta-cohort. **(A)** Kaplan-Meier analysis of OS stratified based on anoikis and ErbB levels; **(B)** Kaplan-Meier analysis of OS stratified based on the combination of anoikis and ErbB level; **(C)** Differential immune cells infiltration analysis between anoikis^high^&ErbB^high^ group and others; **(D)** The NOTCH pathway was identified between anoikis^high^&ErbB^high^ group and others via GSEA analysis; **(E)** Heatmap showed the correlations among anoikis level, ErbB pathway level, NOTCH pathway level and macrophage infiltration in meta-cohort. OS, Overall Survival; GSEA, Gene Set Enrichment Analysis; *: P < 0.05; **: P < 0.01; ***: P < 0.001.

Supplementary Figure 4 The prognostic value of five signature predictors. **(A-E)** Kaplan-Meier analysis of OS stratified based on the expression status of ECT2, CCT2, CDK4, SLC2A1, MARCKSL1 genes in TCGA cohort. **(F)** Univariate Cox regression analysis of signature predictors in TCGA cohort. **(G-K)** Kaplan-Meier analysis of OS stratified based on the expression status of ECT2, CCT2, CDK4, SLC2A1, MARCKSL1 genes in meta-cohort. **(L)** Univariate Cox regression analysis of signature predictors in meta-cohort. OS, Overall Survival; TCGA, The Cancer Genome Atlas.

Supplementary Figure 5 Comparison of AERS and other prognostic signatures in HCC. **(A)** Univariate Cox regression analysis of AERS and 72 published signatures in the TCGA, ICGC, GSE144269, GSE116174, GSE14520 and mate-cohort. **(B-G)** C-index of AERS and 72 published signatures in the TCGA, ICGC, GSE144269, GSE116174, GSE14520 and mate-cohort. AERS, Anoikis&ErbB related signature HCC, Hepatocellular carcinoma; TCGA, The Cancer Genome Atlas; ICGC, International Cancer Genome Construction.

Supplementary Figure 6 The counts **(A)** and intensity **(B)** of ligand receptors between different cell types with poor prognosis is shown by circle plots.

Supplementary Figure 7 Potential mechanism of AERS in sorafenib resistance. **(A)** UMAP plot and bar plots indicating the landscape and proportion of major cell lineages between sorafenib resistant group and the other cells. **(B)** UMAP plot and violin plot shows the distribution of anoikis level, ErbB pathway level, NOTCH pathway level and AERS between sorafenib resistant group and the other cells. **(C)** Pseudotime analysis for the anoikis^high^&ErbB^high^ macrophages with sorafenib resistant. **(D)** Heatmap indicating the activation of major pathways in cell subpopulation with sorafenib resistant. AERS, Anoikis&ErbB related signature HCC, Hepatocellular carcinoma; scRNA-seq, Single-cell RNA sequencing; UMAP, Uniform Manifold Approximation and Projection; ***:P < 0.001.

Supplementary Figure 8 The counts**(A)** and intensity**(B)** of ligand receptors between different cell types with sorafenib resistant is shown by circle plots.

Supplementary Figure 9 The application of AERS in ICIs among phs000452 cohort and Braun-2020 cohort. **(A)** The distribution of the differential AERS between sensitive and resistant groups in the phs000452 cohort. **(B)** Fourfold table between AERS and sorafenib response in the phs000452 cohort. **(C)** ROC curve of AERS to predict ICIs response in the phs000452 cohort. **(D)** Kaplan-Meier curves of OS with different AERS groups in the phs000452 cohort. **(E)** Time-dependent ROC analysis of AERS for predicting OS at 1, 2, and 3 years in the phs000452 cohort. **(F)** The distribution of the differential AERS between sensitive and resistant groups in the Braun-2020 cohort. **(G)** Fourfold table between AERS and ICIs response in the Braun-2020 cohort. **(H)** ROC curve of AERS to predict ICIs response in the Braun-2020 cohort. **(I)** Kaplan-Meier curves of OS with different AERS groups in the Braun-2020 cohort. **(J)** Time-dependent ROC analysis of AERS for predicting OS at 1, 2, and 3 years in the Braun-2020 cohort. ICIs, Immune checkpoint inhibitors; AERS, Anoikis&ErbB related signature; ROC, Receiver operating characteristic; OS, Overall Survival. **p < 0.01; ***p < 0.001.

Supplementary Figure 10 The prognostic value of five signature predictors in an in-house cohort. **(A-E)** Kaplan-Meier curves of OS according to the ECT2, CCT2, CDK4, SLC2A1, MARCKSL1 genes expression. **(F)** Univariate and multivariate Cox regression analysis of signature genes. OS, Overall Survival.

Supplementary Figure 11 Clinicopathological characteristics evaluation by AERS in an in-house cohort. **(A-D)** Boxplot shows the distribution of AERS and the expression of five signature predictors (ECT2, CCT2, CDK4, SLC2A1, and MARCKSL1) between the groups of age, gender, grade and clinical stage. *p < 0.05.
