# Supplementary figures and images for: Integrating anoikis and ErbB signaling insights with machine learning and single-cell analysis for predicting prognosis and immune-targeted therapy outcomes in hepatocellular carcinoma
Source: Front Immunol. 2024 Oct 11;15:1446961. doi: 10.3389/fimmu.2024.1446961 (PMC11502379; doi:10.3389/fimmu.2024.1446961)

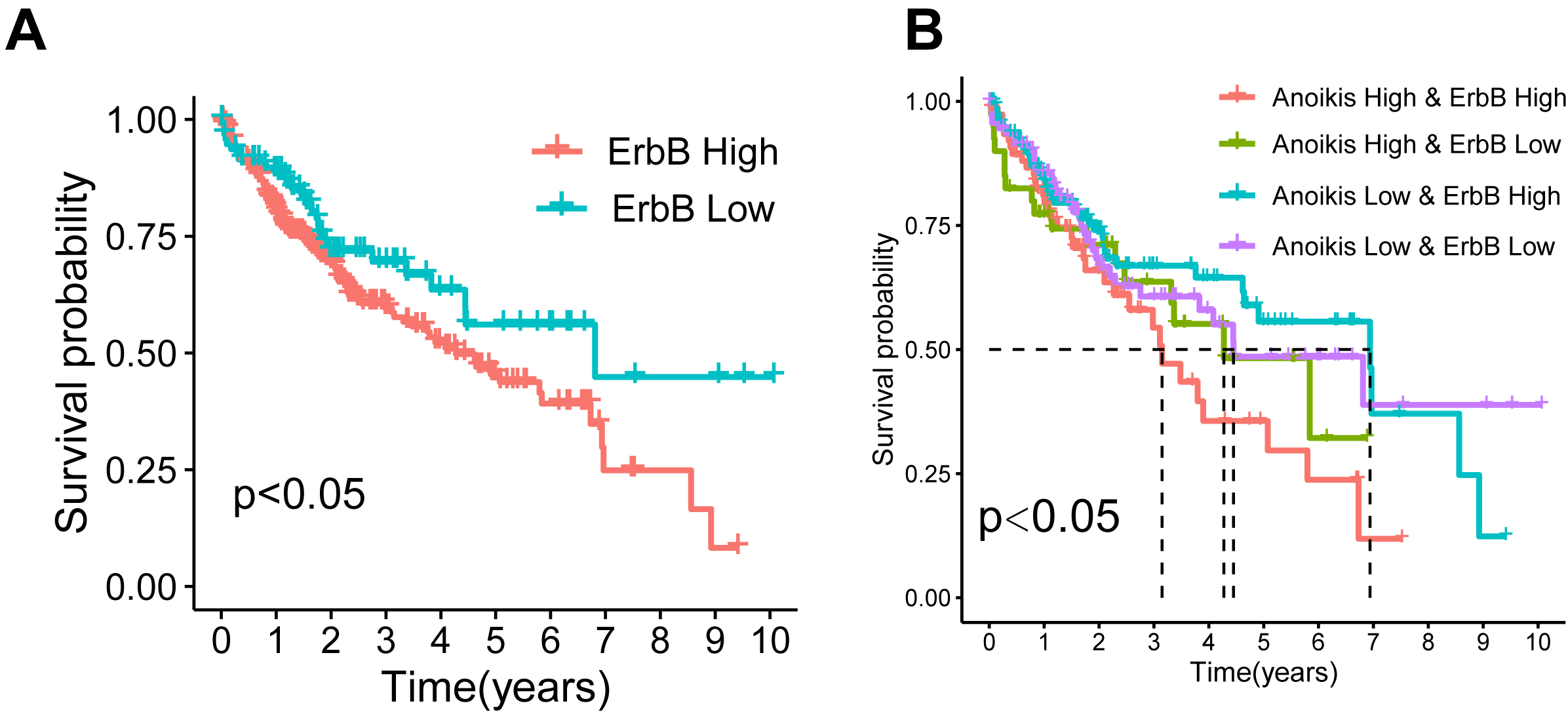

Supplement: Supplementary Figure 1 — Kaplan-Meier analysis of OS stratified based on ssGSEA scores of ErbB level (A), as well as anoikis&ErbB levels (B). [file Image1.tif]

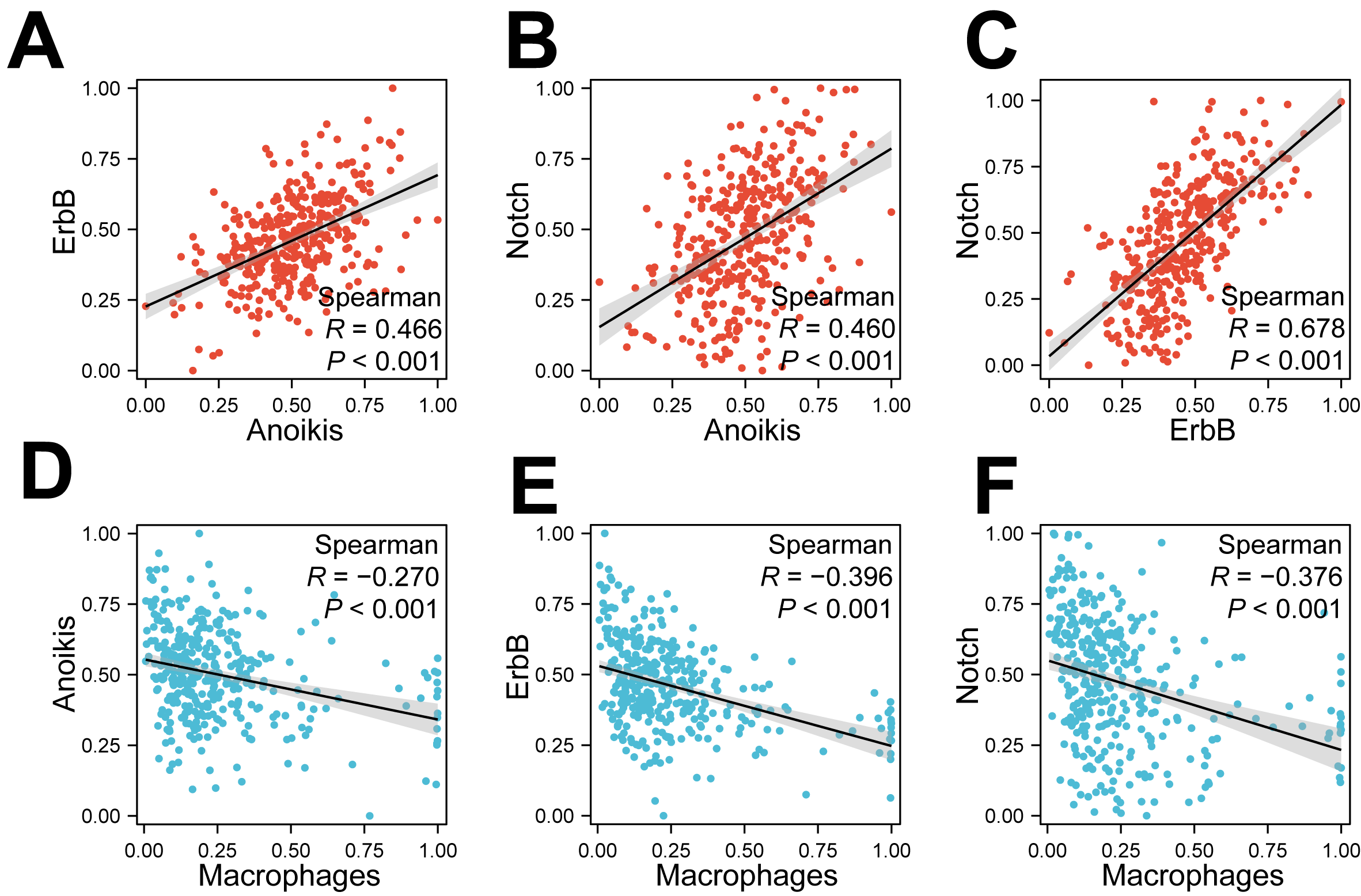

Supplement: Supplementary Figure 2 — (A-F) Scatter plot shows the correlations among anoikis level, ErbB pathway level, NOTCH pathway level and macrophage infiltration. [file Image2.tif]

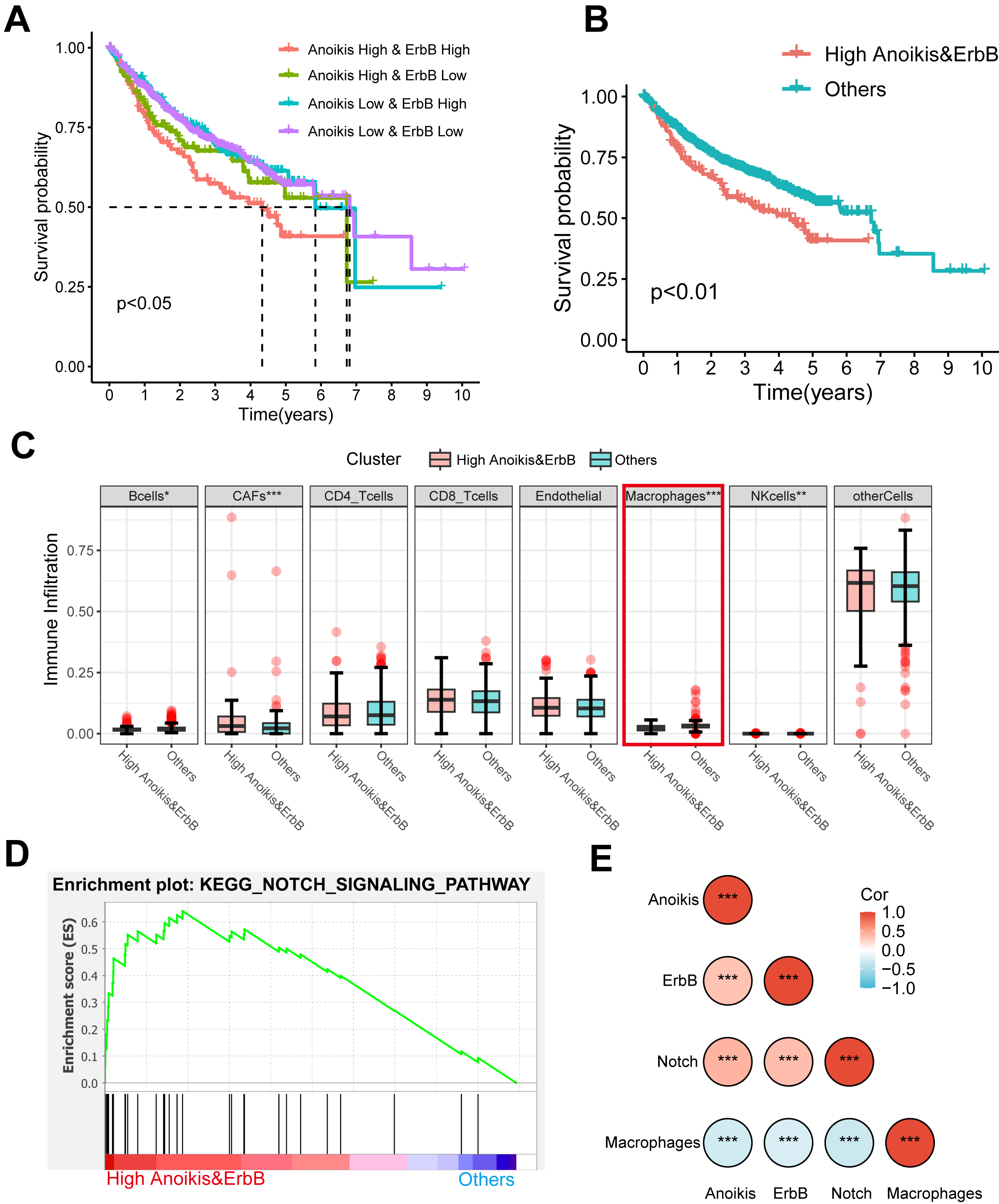

Supplement: Supplementary Figure 3 — The clinical significance and potential mechanism exploration of anoikis levels in meta-cohort. (A) Kaplan-Meier analysis of OS stratified based on anoikis and ErbB levels; (B) Kaplan-Meier analysis of OS stratified based on the combination of anoikis and ErbB level; (C) Differential immune cells infiltration analysis between anoikishigh&ErbBhigh group and others; (D) The NOTCH pathway was identified between anoikishigh&ErbBhigh group and others via GSEA analysis; (E) Heatmap showed the correlations among anoikis level, ErbB pathway level, NOTCH pathway level and macrophage infiltration in meta-cohort. OS, Overall Survival; GSEA, Gene Set Enrichment Analysis; *: P < 0.05; **: P < 0.01; ***: P < 0.001. [file Image3.tif]

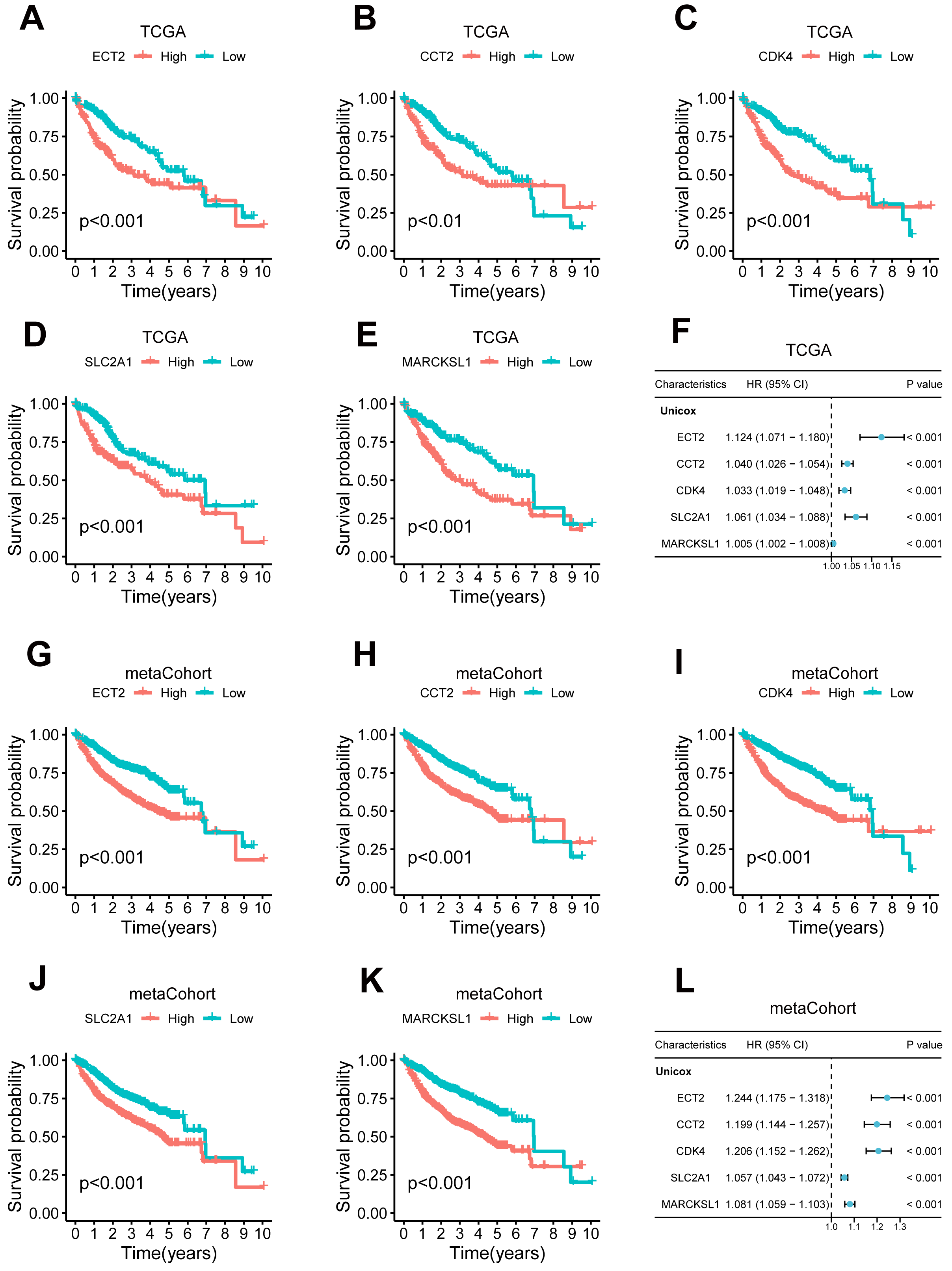

Supplement: Supplementary Figure 4 — The prognostic value of five signature predictors. (A-E) Kaplan-Meier analysis of OS stratified based on the expression status of ECT2, CCT2, CDK4, SLC2A1, MARCKSL1 genes in TCGA cohort. (F) Univariate Cox regression analysis of signature predictors in TCGA cohort. (G-K) Kaplan-Meier analysis of OS stratified based on the expression status of ECT2, CCT2, CDK4, SLC2A1, MARCKSL1 genes in meta-cohort. (L) Univariate Cox regression analysis of signature predictors in meta-cohort. OS, Overall Survival; TCGA, The Cancer Genome Atlas. [file Image4.tif]

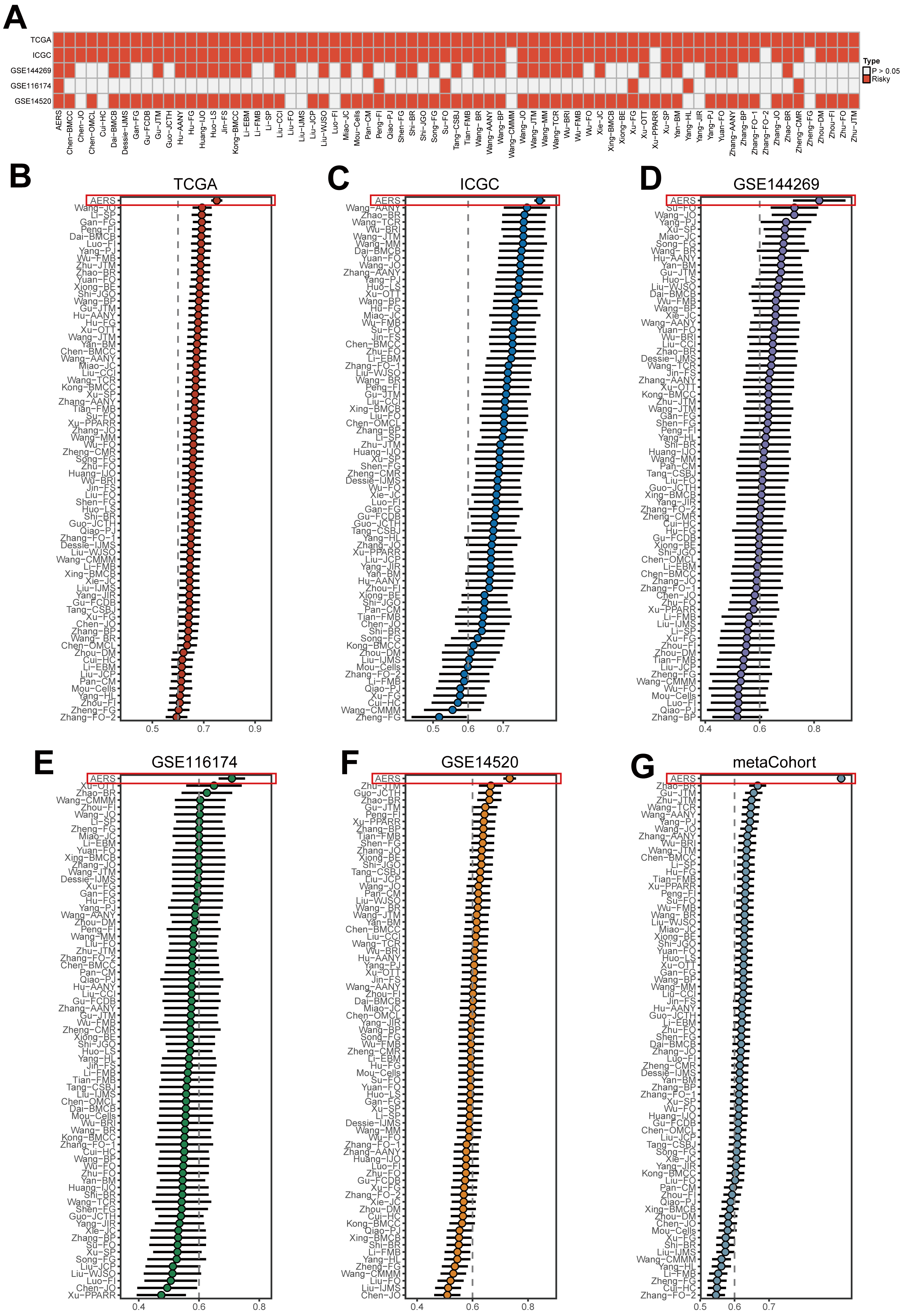

Supplement: Supplementary Figure 5 — Comparison of AERS and other prognostic signatures in HCC. (A) Univariate Cox regression analysis of AERS and 72 published signatures in the TCGA, ICGC, GSE144269, GSE116174, GSE14520 and mate-cohort. (B-G) C-index of AERS and 72 published signatures in the TCGA, ICGC, GSE144269, GSE116174, GSE14520 and mate-cohort. AERS, Anoikis&ErbB related signature HCC, Hepatocellular carcinoma; TCGA, The Cancer Genome Atlas; ICGC, International Cancer Genome Construction. [file Image5.tif]

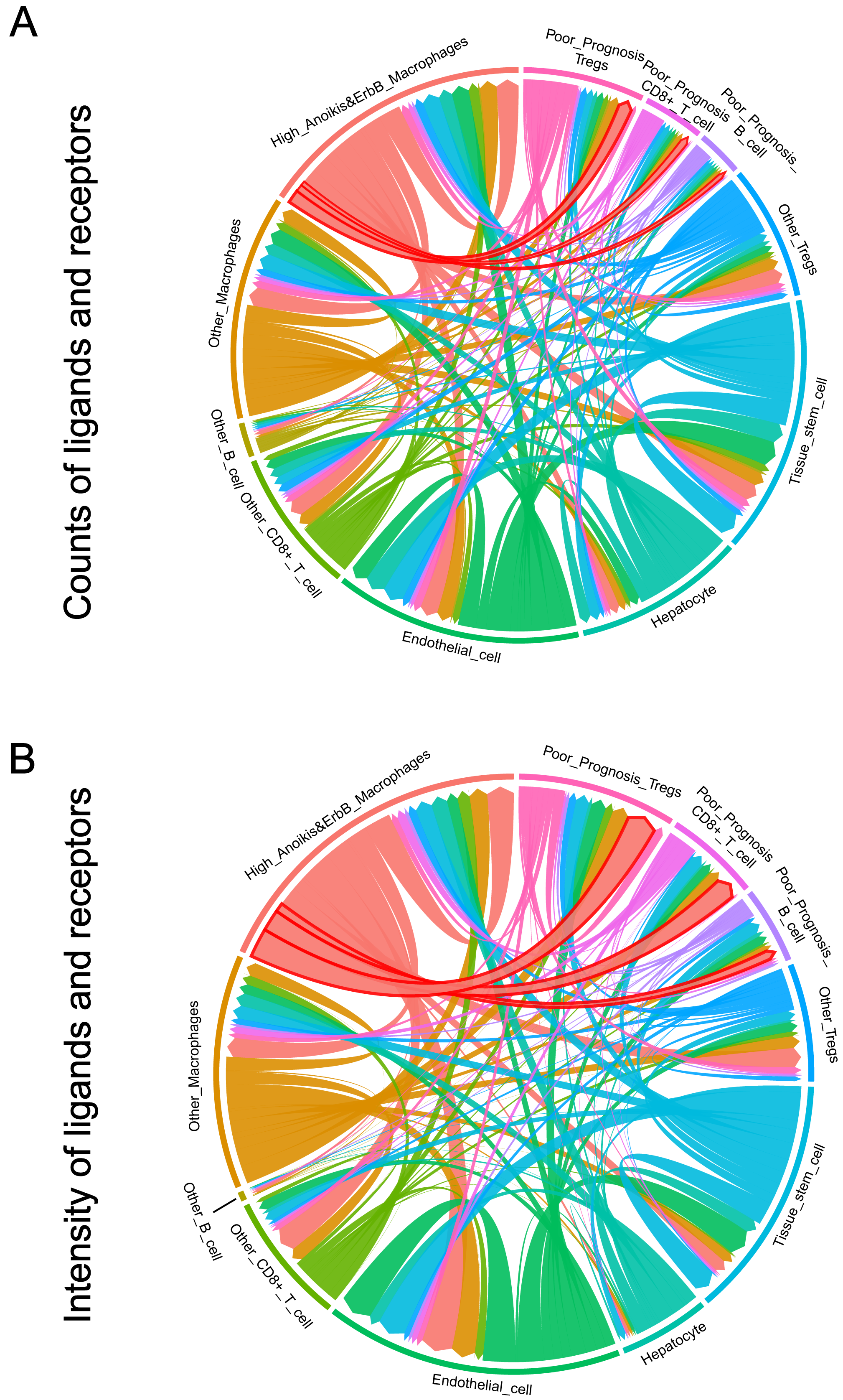

Supplement: Supplementary Figure 6 — The counts (A) and intensity (B) of ligand receptors between different cell types with poor prognosis is shown by circle plots. [file Image6.tif]

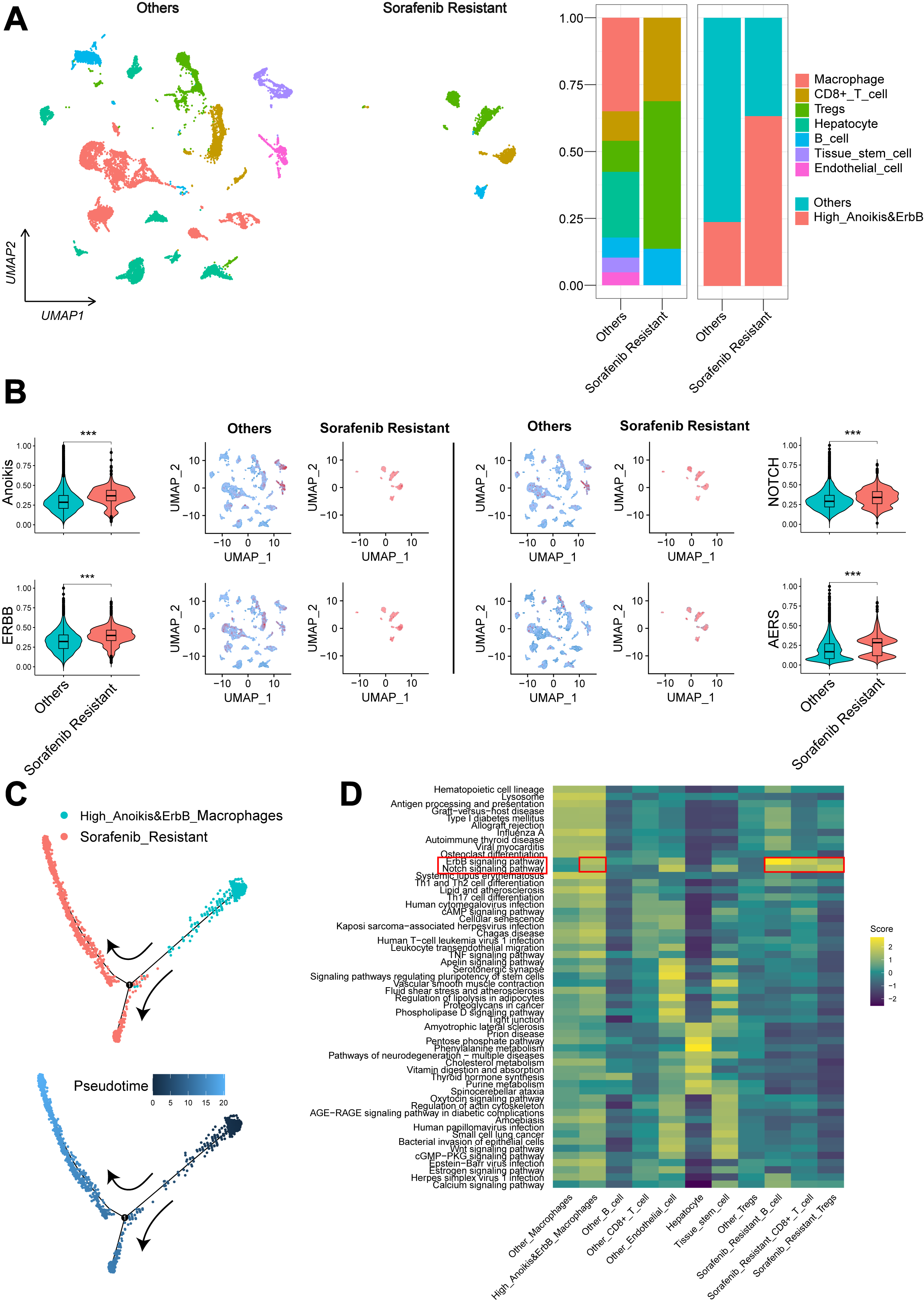

Supplement: Supplementary Figure 7 — Potential mechanism of AERS in sorafenib resistance. (A) UMAP plot and bar plots indicating the landscape and proportion of major cell lineages between sorafenib resistant group and the other cells. (B) UMAP plot and violin plot shows the distribution of anoikis level, ErbB pathway level, NOTCH pathway level and AERS between sorafenib resistant group and the other cells. (C) Pseudotime analysis for the anoikishigh&ErbBhigh macrophages with sorafenib resistant. (D) Heatmap indicating the activation of major pathways in cell subpopulation with sorafenib resistant. AERS, Anoikis&ErbB related signature HCC, Hepatocellular carcinoma; scRNA-seq, Single-cell RNA sequencing; UMAP, Uniform Manifold Approximation and Projection; ***:P < 0.001. [file Image7.tif]

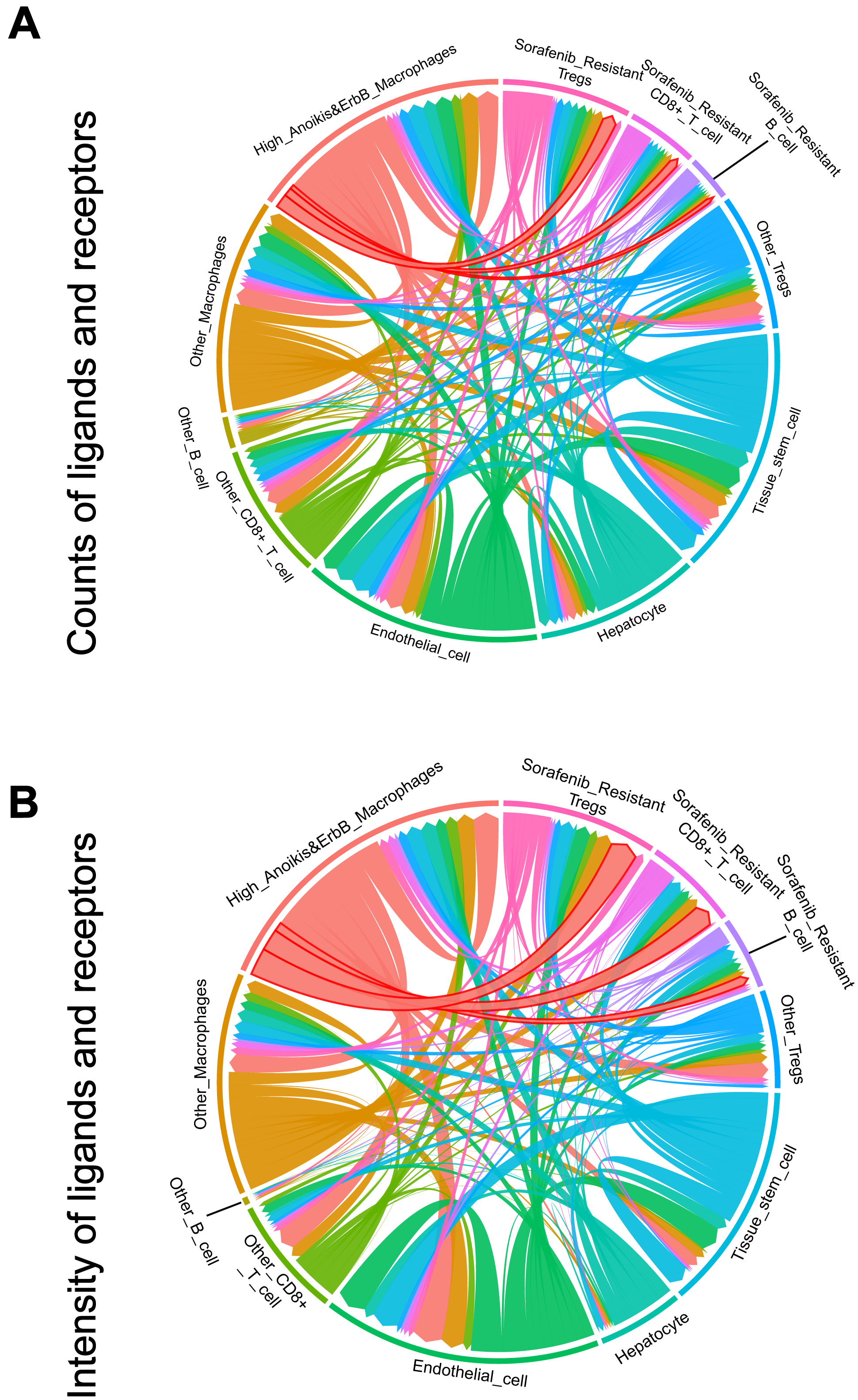

Supplement: Supplementary Figure 8 — The counts(A) and intensity(B) of ligand receptors between different cell types with sorafenib resistant is shown by circle plots. [file Image8.tif]

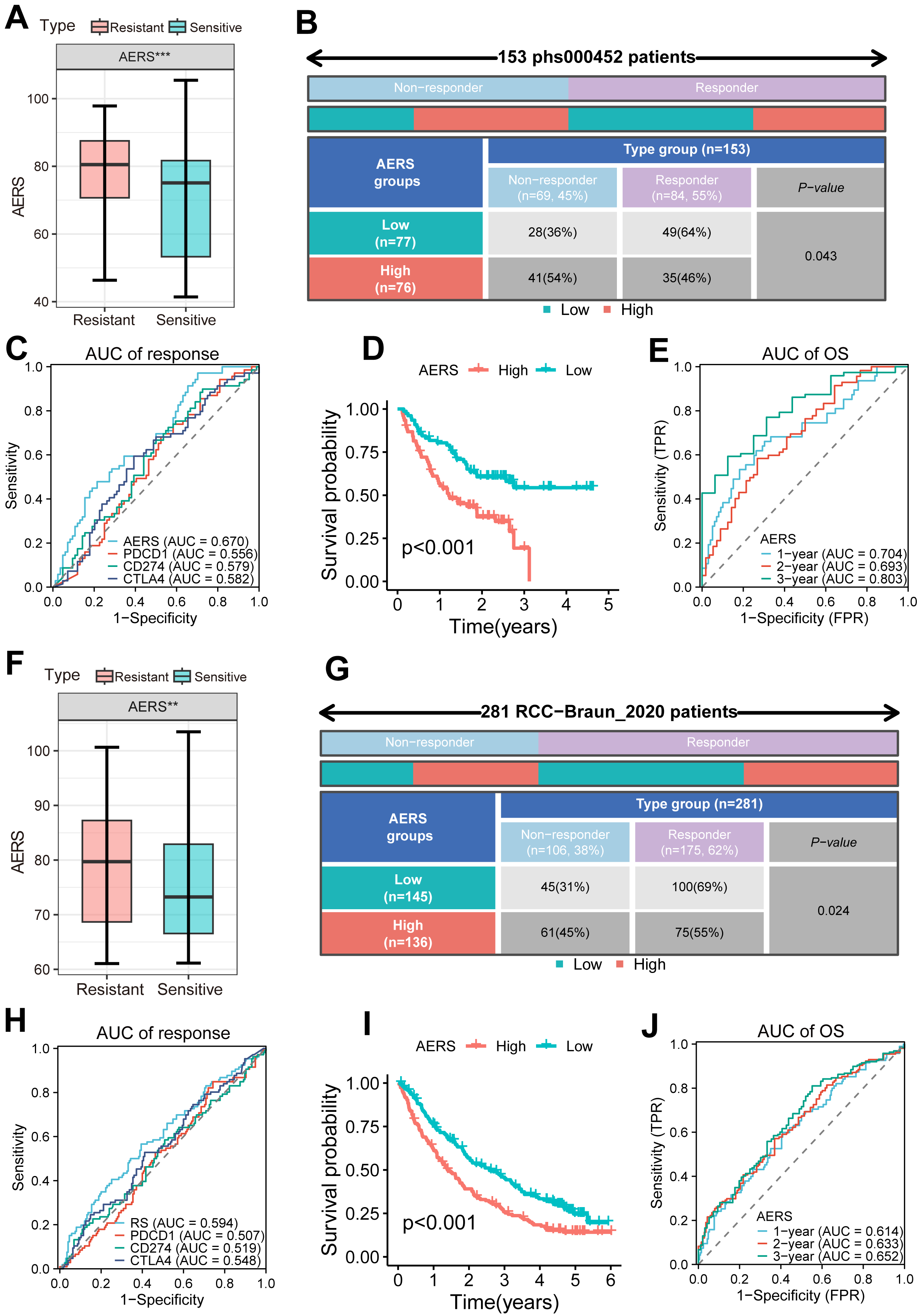

Supplement: Supplementary Figure 9 — The application of AERS in ICIs among phs000452 cohort and Braun-2020 cohort. (A) The distribution of the differential AERS between sensitive and resistant groups in the phs000452 cohort. (B) Fourfold table between AERS and sorafenib response in the phs000452 cohort. (C) ROC curve of AERS to predict ICIs response in the phs000452 cohort. (D) Kaplan-Meier curves of OS with different AERS groups in the phs000452 cohort. (E) Time-dependent ROC analysis of AERS for predicting OS at 1, 2, and 3 years in the phs000452 cohort. (F) The distribution of the differential AERS between sensitive and resistant groups in the Braun-2020 cohort. (G) Fourfold table between AERS and ICIs response in the Braun-2020 cohort. (H) ROC curve of AERS to predict ICIs response in the Braun-2020 cohort. (I) Kaplan-Meier curves of OS with different AERS groups in the Braun-2020 cohort. (J) Time-dependent ROC analysis of AERS for predicting OS at 1, 2, and 3 years in the Braun-2020 cohort. ICIs, Immune checkpoint inhibitors; AERS, Anoikis&ErbB related signature; ROC, Receiver operating characteristic; OS, Overall Survival. **p < 0.01; ***p < 0.001. [file Image9.tif]

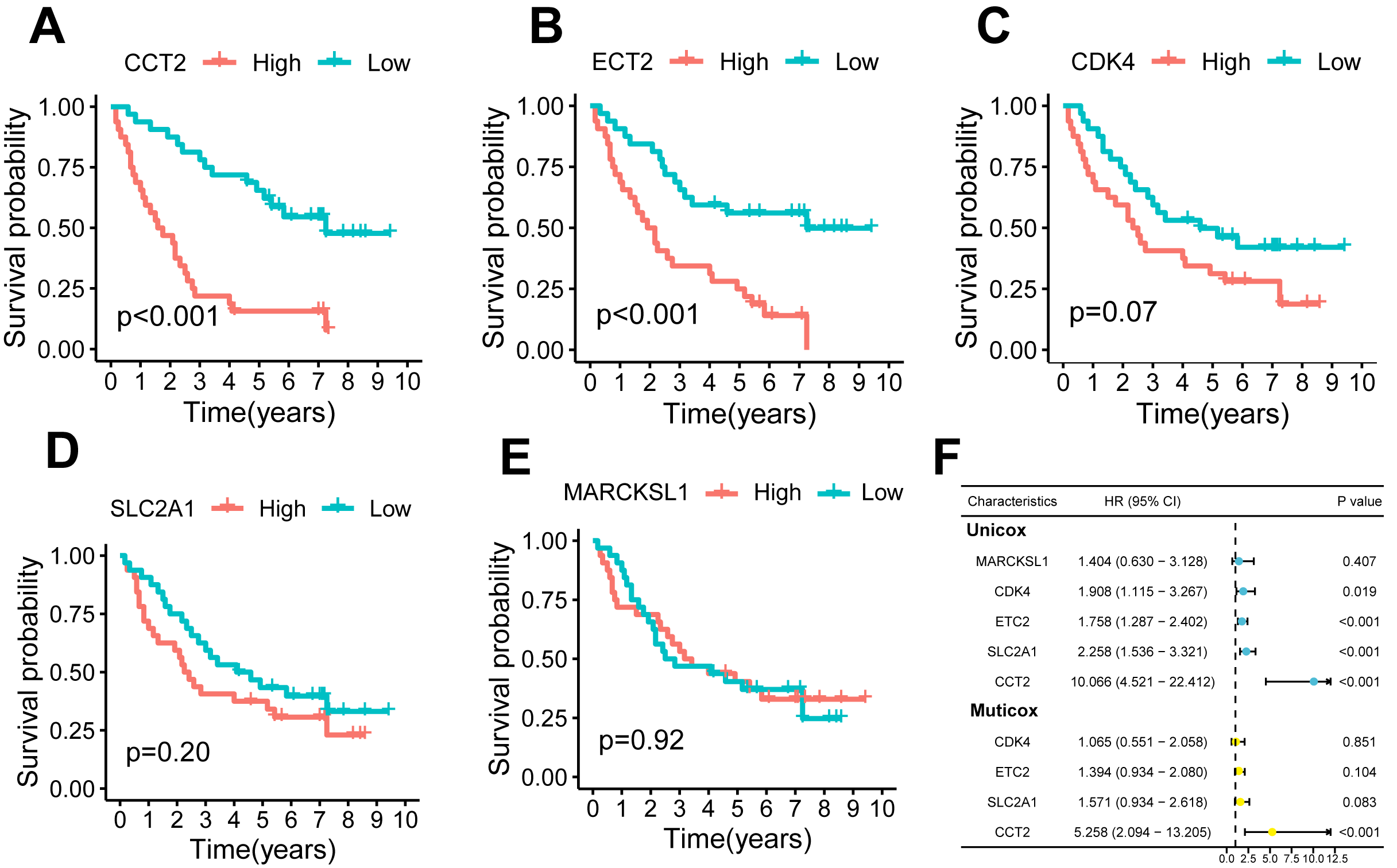

Supplement: Supplementary Figure 10 — The prognostic value of five signature predictors in an in-house cohort. (A-E) Kaplan-Meier curves of OS according to the ECT2, CCT2, CDK4, SLC2A1, MARCKSL1 genes expression. (F) Univariate and multivariate Cox regression analysis of signature genes. OS, Overall Survival. [file Image10.tif]

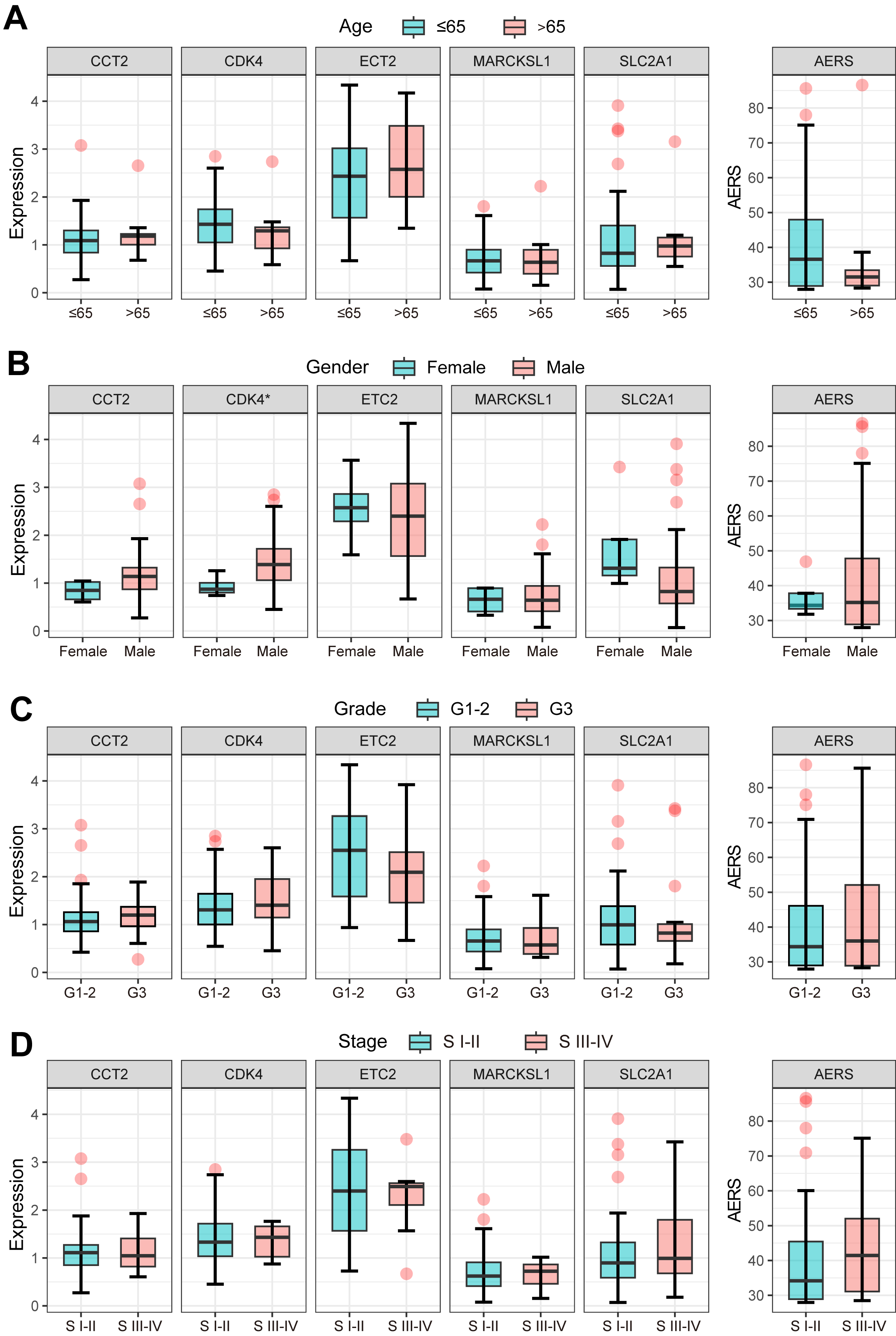

Supplement: Supplementary Figure 11 — Clinicopathological characteristics evaluation by AERS in an in-house cohort. (A-D) Boxplot shows the distribution of AERS and the expression of five signature predictors (ECT2, CCT2, CDK4, SLC2A1, and MARCKSL1) between the groups of age, gender, grade and clinical stage. *p < 0.05. [file Image11.tif]
